# Brawn and Brainpower: Acute Resistance Exercise Improves Behavioral and Neuroelectric Measures of Executive Function

**DOI:** 10.1111/psyp.70171

**Published:** 2025-10-30

**Authors:** Nicholas W. Baumgartner, Michael D. Belbis, Kyoungmin Noh, Daniel M. Hirai, Steve Amireault, Shih‐Chun Kao

**Affiliations:** ^1^ Department of Health and Kinesiology Purdue University West Lafayette Indiana USA; ^2^ RUSH Alzheimer's Disease Center, RUSH University Medical Center Chicago Illinois USA; ^3^ Department of Kinesiology and Physical Education Northern Illinois University DeKalb Illinois USA

**Keywords:** cognitive enhancement, inhibitory control, mediation analysis, processing speed, systolic blood pressure, working memory

## Abstract

Acute resistance exercise (RE) is emerging as a promising strategy to improve executive function, yet the underlying mechanisms remain unclear. In this study, 121 participants (aged 18–50) were randomly assigned to either a moderate‐intensity RE or rest intervention using a between‐subjects design. We examined the effects of acute RE on behavioral and neuroelectric measures of executive function during a modified Flanker task and an N‐back task, and collected lactate and blood pressure to explore possible physiological mechanisms. Results showed that following acute RE, blood lactate (*d* = 2.06) and systolic blood pressure (*d* = 0.99) significantly increased. Improvements in executive function were similarly observed following acute RE, including faster processing speed during inhibitory control (*d* = 0.37) and working memory (*d* = 0.46), and decreased P3 latency during inhibitory control (*d* = 0.41). Moreover, exploratory mediation analyses revealed that systolic blood pressure mediated the effect of RE on response time during both the Flanker (−10.73 ± 4.99, 95% CI = −21.50 to −1.52) and N‐back (−28.20 ± 12.38, 95% CI = −53.22 to −5.37) tasks. Overall, these findings provide evidence that acute RE enhances neuroelectric and behavioral markers of inhibitory control and working memory performance and highlight the potential for systolic pressure as a mechanism through which acute RE influences cognition.

## IMPACT STATEMENT

Building upon established aerobic exercise research, the current study highlights the potential of acute resistance exercise (RE) for enhancing executive function, including faster processing speeds during inhibitory control and working memory. Novel investigations into the underlying neuroelectric correlates of attention and conflict monitoring revealed a selective RE‐induced speed and efficiency gain for inhibitory control but not working memory. RE‐induced changes in systolic blood pressure mediated the effects on executive function, indicating a potential physiological mechanism behind improved executive function. These findings advance the understanding of exercise‐induced cognitive improvements, and suggest new avenues for cognitive health interventions.

## 1 Introduction

Resistance exercise (RE) has emerged as a critical component of health, playing a key role in regulating blood glucose (Taha et al. 2022), reducing obesity (Strasser and Schobersberger 2010), and lowering blood pressure (Correia et al. 2023). Although the benefits of acute aerobic exercise on executive functions are well documented (Huang et al. 2022; Ishihara et al. 2021; Ludyga et al. 2016), few studies have investigated acute RE, despite evidence RE impacts the neurophysiological responses underlying executive function (Baumgartner and Kao 2024; Chia‐Liang Tsai et al. 2014; Wu et al. 2019). Although few studies have shown acute RE improves aspects of executive function, including problem‐solving (Chang et al. 2011), inhibitory control (Chang et al. 2017, 2014; Tsai et al. 2014), and cognitive flexibility (Wu et al. 2019). Meta‐analyses indicate a positive effect of acute RE, compared to a no‐exercise control, on inhibitory control (standardized mean difference (SMD): 0.73, 95% CI 0.21–1.26), while working memory (SMD: 0.35, 95% CI −0.05 to 0.70) has shown mixed results (Wilke et al. 2019). Although this body of literature is still emerging, these effect sizes of RE are substantial compared to the effect of acute aerobic exercise on executive function in young adults (Hedges' *g* = 0.20, 95% CI 0.07–0.34) (Ludyga et al. 2016), highlighting the potential of acute RE to enhance executive functions.

Electroencephalography (EEG) and event‐related potentials (ERPs) offer a precise measure of brain activity related to executive function. Notably, the P3 (or P300, P3b) component—the positive voltage deflection occurring 300 to 700 ms post‐stimulus onset—is commonly investigated concerning the acute effect of exercise on executive function. P3 amplitude represents the magnitude of neural inhibition used to facilitate the updating of the mental representation of working memory load or attentional resource allocation, while P3 latency indexes stimulus categorization speed and cognitive processing efficiency, proportional to the time required to detect, evaluate, and respond to a target stimulus (Donchin 1981; Kramer et al. 1983; Magliero et al. 1984; Polich 2007; Verleger 1997). Past works have established a small‐to‐moderate effect of acute exercise on increasing P3 amplitude (*g* = 0.32, 95% CI = 0.21–0.42) and decreasing P3 latency (*g* = 0.15, 95% CI = 0.04–0.25) during various executive function tasks, with RE exerting the largest effect on P3 amplitude (*g* = 0.47, 95% CI = 0.13–0.81) (Kao et al. 2022). Although the interpretation of P3 can depend on the task used to elicit ERP, exercise‐induced changes in P3 amplitude and latency are not moderated by cognitive domains (Kao et al. 2022) and have been frequently used as complementary measures to explain enhanced executive function performance following exercise. Specifically, increased P3 amplitude and decreased P3 latency following exercise have been interpreted as the effective allocation of attention and faster processing speed to support successful performance during tasks requiring inhibitory control and working memory (Kao et al. 2022). P3 amplitude and latency changes have been linked with elevated arousal (Lambourne and Tomporowski 2010) through an activated locus coeruleus norepinephrine (LC‐NE) system in response to exercise and in support of post‐exercise executive function (McMorris 2016). The N2, another commonly examined ERP component, is defined as the negative deflection in voltage that occurs between 200 and 400 ms following stimulus onset. This component is involved in conflict monitoring, especially the inhibition component of cognitive control, with larger (more negative) amplitude reflecting a greater level of conflict detection (van Veen and Carter 2002). While some studies have reported a positive effect of acute AE on the N2 component (Aly and Kojima 2020; Ligeza et al. 2018; Winneke et al. 2019), most report no effect (Gusatovic et al. 2022) and the effect of acute RE on N2 remains largely unexplored.

The mechanisms linking RE with cognition are not well understood, but physiological responses to acute RE have been associated with enhanced executive function (Chia‐Liang Tsai et al. 2014). Acute exercise is theorized to improve cognitive function through increased arousal (Lambourne and Tomporowski 2010), wherein exercise alters systems that influence how mental resources are allocated to cognitive functions (Audiffren et al. 2009), reflected in altered cardiovascular (i.e., blood pressure and heart rate (HR)), cortical (e.g., changes in ERPs), and peripheral (i.e., lactate) responses. Additionally, lactate released during exercise—once considered a metabolic waste product—serves as an energy substrate in the brain (Barros et al. 2009), supporting up to 75% of the brain's oxidative metabolism (Bouzier‐Sore et al. 2006) and acting as an additional fuel source (Hashimoto et al. 2021; Nalbandian and Takeda 2016; Quistorff et al. 2008). Understanding the pathways through which RE influences cognition is essential for optimizing exercise prescription to enhance executive functions. Identifying specific mechanisms allows scientists and clinicians to refine exercise parameters (e.g., intensity, duration, type) to target key pathways, maximizing cognitive benefits.

This study had two objectives: first, to evaluate the effect of acute RE on executive function, and second, to explore potential mechanisms underlying these cognitive changes. It was hypothesized that acute RE would improve both behavioral (faster, more accurate performance) and neuroelectric indices (increased amplitude and decreased latency) of executive function. Additionally, it was hypothesized that changes in physiological outcomes (lactate and blood pressure) would mediate the RE‐related enhancement of executive functions.

## 2 Methods

### 2.1 Subjects

One hundred twenty‐one healthy adults (18–50 years old) completed this between‐subjects randomized controlled trial. All subjects reported normal or correct‐to‐normal vision (e.g., 20/20), no contraindication for exercise determined by the Physical Activity Readiness Questionnaire (Thomas et al. 1992), no history of neurological, cardiovascular, or respiratory disease, and did not take medication that could influence central nervous system function (e.g., anti‐psychotic, anti‐depressant, anti‐anxiety, or ADD/ADHD medications). Written informed consent was obtained, and the study was approved by the Institutional Review Board of Purdue University (IRB‐2022‐307). To ensure baseline exercise familiarity, subjects were required to have recreationally exercised at least 20 min on 2+ days per week over the past year. Subject characteristics and baseline group differences are detailed in Table 1.

**TABLE 1:** Subject characteristics.

	Total (*n* = 121)	RE group (*n* = 62)	Rest group (*n* = 59)	Group difference
Age (years)	26.69 (7.78)	26.89 (8.02)	26.47 (7.58)	0.77
Female (*n*, %)	50, 41%	23, 37%	27, 46%	0.61
Height (cm)	171.03 (9.65)	172.36 (8.36)	169.64 (10.74)	0.12
Weight (kg)	73.70 (14.87)	75.98 (14.76)	71.31 (14.73)	0.08
BMI (kg/m^2^)	25.05 (3.95)	25.47 (4.23)	24.62 (6.62)	0.24
Time between visits (days)	10.96 (12.40)	9.94 (9.38)	12.03 (14.94)	0.35
Weight lifted–1RM (kg)				
Chest press	64.03 (28.83)	67.60 (30.00)	60.29 (27.31)	0.16
Lat pulldown	80.10 (32.56)	84.47 (34.32)	75.50 (30.22)	0.13
Bicep curl	15.94 (6.12)	16.66 (6.15)	15.18 (6.04)	0.18
Leg press	167.33 (71.20)	176.79 (76.70)	157.38 (64.07)	0.14
Triceps extension	25.82 (8.85)	**27.53 (9.25)**	**24.03 (8.09)**	0.03*
Leg extension	76.66 (28.88)	80.57 (30.94)	72.55 (26.18)	0.13
Handgrip strength (kg)	43.50 (11.89)	44.37 (10.94)	42.59 (12.85)	0.41
V̇O2max (mL/kg/min)	43.60 (8.64)	44.73 (8.11)	42.42 (9.08)	0.14

*Note:* Values are mean ± SD at baseline unless otherwise noted; Group difference column represents the *p*‐value (two‐tailed) from an independent samples *t*‐test for mean difference between RE and rest group, significant group differences are bolded for clarity. (* = *p* < 0.05).

Subjects were randomly assigned to either the RE or rest condition based on the order they contacted the lab. A complete list of subject IDs, preassigned to all counterbalance conditions, was generated prior to the study. The list order was randomized using a random number generator (random.org), and IDs were assigned sequentially as subjects completed the screening form.

### 2.2 Procedures

**FIGURE 1:**
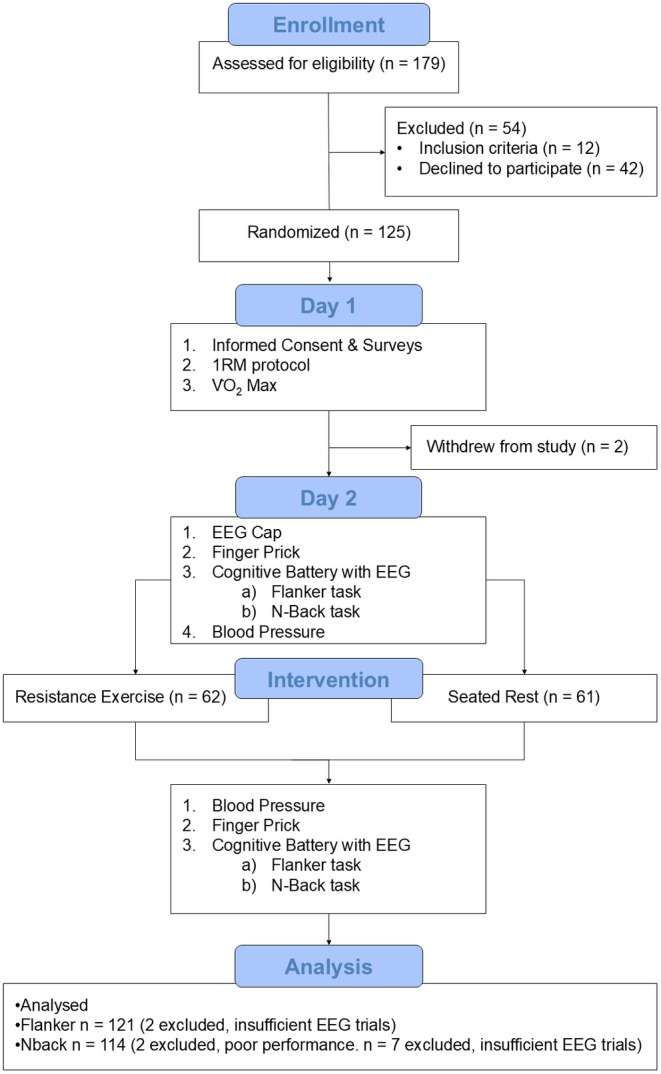
Experimental flow diagram: The tasks and procedures from initial contact to data analysis, using a between‐subjects randomized controlled trial design.

Laboratory visits were scheduled 7 days apart when possible, with a minimum interval of 2 days. Subjects refrained from vigorous activity and caffeinated drinks for 12+ hours and alcohol for 24+ h before each visit. Day 1: subjects completed an informed consent, a demographic survey, a 1RM protocol, and a graded maximal exercise test (V̇O2_max_; detailed in Supporting Information S4). Day 2: subjects were fitted with an EEG cap, underwent a finger‐prick blood collection, then completed executive function tasks while EEG data were recorded. Next, seated blood pressure and HR were measured, followed by either an RE or seated video‐watching rest intervention. Ratings of perceived exertion (RPE) and HR were measured after each set (every 3–3.5 min). Afterward, seated blood pressure and HR were measured again, followed by a second finger prick. Subjects then repeated the executive function tasks and EEG recording. A detailed flowchart of the study design is provided in Figure 1.

### 2.3 Executive Function

Two components of executive function were assessed in a fixed order: inhibitory control using a modified Eriksen Flanker Task (Eriksen and Eriksen 1974) followed by working memory using a 2‐back task (Kao et al. 2020; Kirchner 1958). The Flanker Task presented five 2.5 cm tall white arrows (40° visual angle), spaced 8 mm apart, displayed centrally on a black background for 200 ms with a 1000 ms response window. A variable inter‐stimulus interval (ISI) of 1000, 1200, or 1400 ms was used. Subjects responded as quickly and accurately as possible with a left or right thumb press on a response pad (Current Designs Inc., Philadelphia, PA, USA) to indicate the direction of the centrally presented target arrow, flanked by congruent (<<<<< or >>>>>) or incongruent (<<> < < or > > <>>) arrows. After instructions, subjects completed 12 practice trials followed by two blocks of 96 randomized trials, with equiprobable congruency and directionality.

For the 2‐back task, six equiprobable colored shapes were displayed one at a time on a black background. Each shape was presented for 200 ms, followed by a fixed ISI of 2000 ms and a 1500 ms response window after stimulus onset. Subjects responded as quickly and accurately as possible with a right thumb press on a response pad (Current Designs Inc., Philadelphia, PA, USA) when the current stimulus matched the one from two steps earlier (target) and with a left thumb press when it did not (nontarget). After instructions, subjects completed 20 practice trials followed by two blocks of 70 trials (24 targets and 46 nontargets) in a pre‐randomized sequence. Two versions of the task were counterbalanced, with subjects exposed to different versions before and after the intervention. Task examples are provided in the supplemental materials (Figure S1).

### 2.4 One‐Rep Max (1RM) Protocol

Muscle strength was assessed using a 1RM protocol as previously described (Baumgartner et al. 2025). Briefly, following a warm‐up, subjects attempted a single repetition at ~100% of their estimated 1RM. If successful, the load was incrementally increased by a trained experimenter and the new weight was attempted; if unsuccessful, weight was decreased, and another attempt was made. Subjects were allowed a maximum of three attempts per exercise, with 3–5 min of rest between attempts. If 1RM was not achieved after the second attempt, a trained experimenter estimated a predicted 1RM weight, and subjects completed as many reps as possible while maintaining proper technique. In these cases, 1RM was estimated using the National Strength and Conditioning Association training load chart (Baechle et al. 2008; Landers 1984). Six exercises (chest press, latissimus dorsi pulldown, dumbbell bicep curl, leg press, cable triceps extension, and leg extension) were tested consecutively. The heaviest weight successfully lifted for each exercise was recorded and subsequently used to personalize the RE intervention.

### 2.5 Acute Interventions

The RE condition lasted ~42 min, beginning with a 2‐min warm‐up followed by two sets of 10 repetitions for each exercise (chest press, latissimus dorsi pulldown, dumbbell bicep curl, leg press, cable triceps extension, and leg extension). Intensity was set at 65%–75% of each subject's 1RM, with rest periods ranging from 60 to 120 s between sets and 120–180 s between exercises. In the rest condition, subjects were seated in the same private area as the RE condition and watched a 42‐min video of a sex‐matched adult performing the RE protocol. The video mimicked the RE condition in timing, social engagement, and environment, with the goal of creating a visual stimulus with similar emotional and cognitive engagement to the RE condition. HR (mean ± SD; RE: 122.38 ± 13.93 bpm; rest: 67.69 ± 5.14) and RPE (RE: 15.44 ± 1.70; rest: 6.10 ± 0.52) were recorded after each exercise during RE and at corresponding time intervals during rest.

### 2.6 Blood Measures

Finger‐prick blood samples (5–25 μL) were obtained before and after the intervention using a lancet (Nova Biomedical, Safety 23G Lancets; Waltham, MA). The second droplet was collected on a lactate testing strip (Sport Resource Group, Lactate Plus Test Strips; Minneapolis, MN) and analyzed with a handheld blood lactate meter (Sport Resource Group, Lactate Plus Meter; Minneapolis, MN). This device demonstrates high accuracy and reliability, and shows a strong correlation (*r* = 0.94) to laboratory‐grade blood gas analyzers (Tanner et al. 2010). Brachial artery blood pressure was measured immediately before and after the intervention with an automatic blood pressure cuff (Lazle, Blood Pressure Monitor HA101; Sheridan, WY). Subjects were seated for at least 5 min before measurements were taken, and the cuff was applied snugly to the bare upper arm following manufacturer's instructions. During the measurement, subjects were instructed to remain quiet and breathe normally. Measurements were repeated if the initial measurement fell outside of the normal range or appeared abnormal.

### 2.7 EEG Assessment

EEG activity was recorded from 64 electrode sites using the international 10–10 system on a Quikcap (Compumedics, Charlotte, NC). Recordings were referenced to averaged mastoids and the mid‐frontal site served as the ground electrode. Electrode impedance was kept below 10kΩ, and electrodes were placed above and below the left orbit to monitor electro‐oculographic (EOG) activity. Continuous data was digitized at 1000 Hz and amplified 500 times with a DC to 70 Hz filter using a Synamps RT amplifier (Compumedics; Charlotte, NC). MATLAB R2017a (Mathworks Inc.; Natick, MA), EEGLAB toolbox (version 14.1.1b) (Delorme and Makeig 2004), and ERPLAB toolbox (version 6.1.4) (Lopez‐Calderon and Luck 2014) were used for offline data processing. Continuous data was corrected offline for EOG artifacts using independent component analysis (ICA) (Pontifex et al. 2017) and segmented into epochs from −200 to 1000 ms, which were baseline corrected using the −200 to 0 ms pre‐stimulus period and filtered using a zero phase‐shift 0.1–30 Hz (24 dB/oct) bandpass filter (IIR Butterworth). Epochs with response errors or artifacts exceeding ±100 μV were rejected.

**FIGURE 2:**
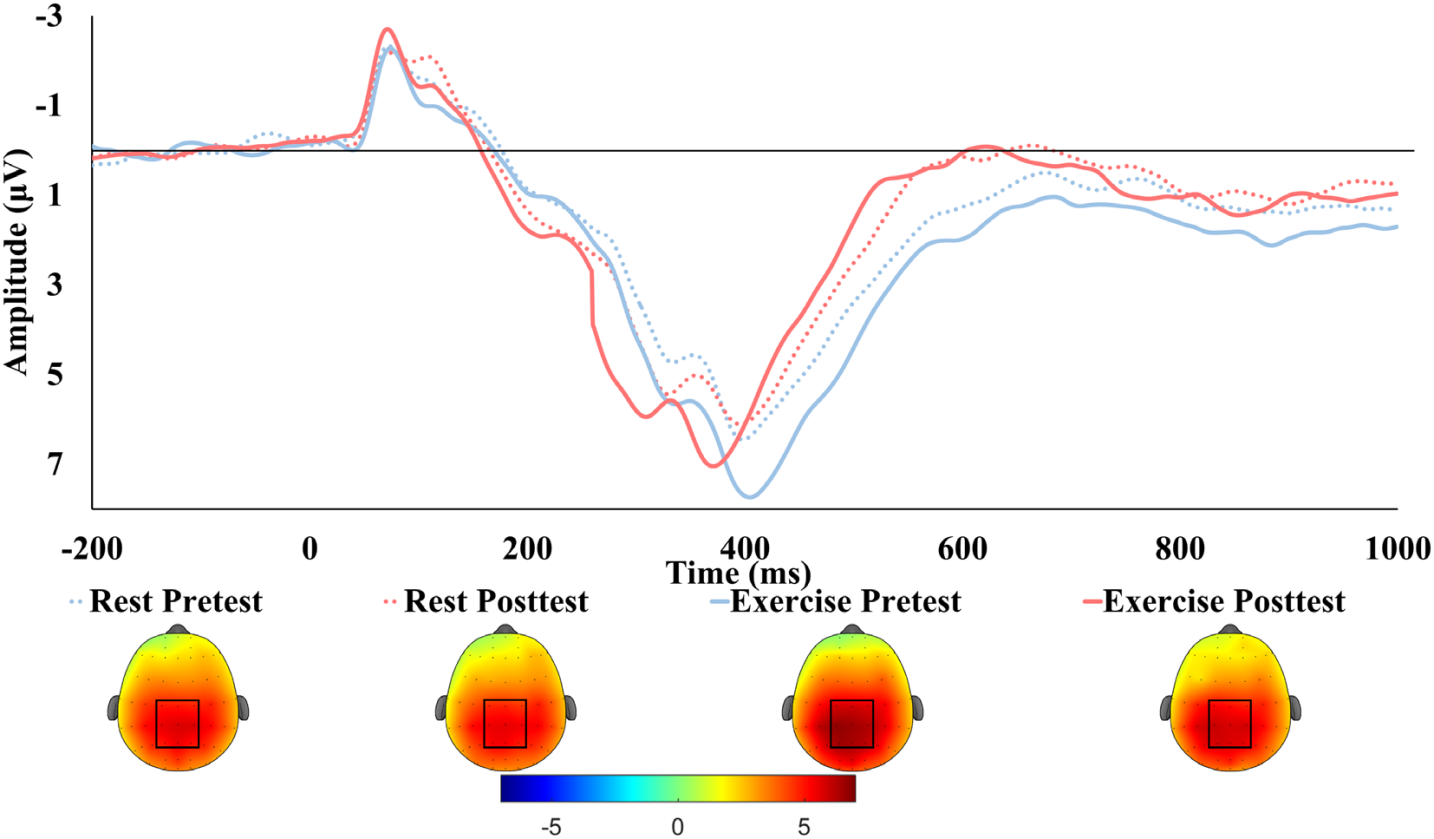
Grand average ERP waveforms during the Flanker task averaged across congruent and incongruent trials. Results showed that while there was no significant change in P3 amplitude from pretest to posttest for either group, following RE P3 latency significantly decreased (solid blue to solid red line) compared to no change following rest (dotted blue to dotted red line). ERP Topoplots averaged across congruent and incongruent trials, using the mean amplitude from 300 to 500 ms are presented. The ROI used in analysis (CPZ, CP1, CP2, PZ, P1, P2, POZ, PO3, & PO4 electrodes) is designated by a square.

**FIGURE 3:**
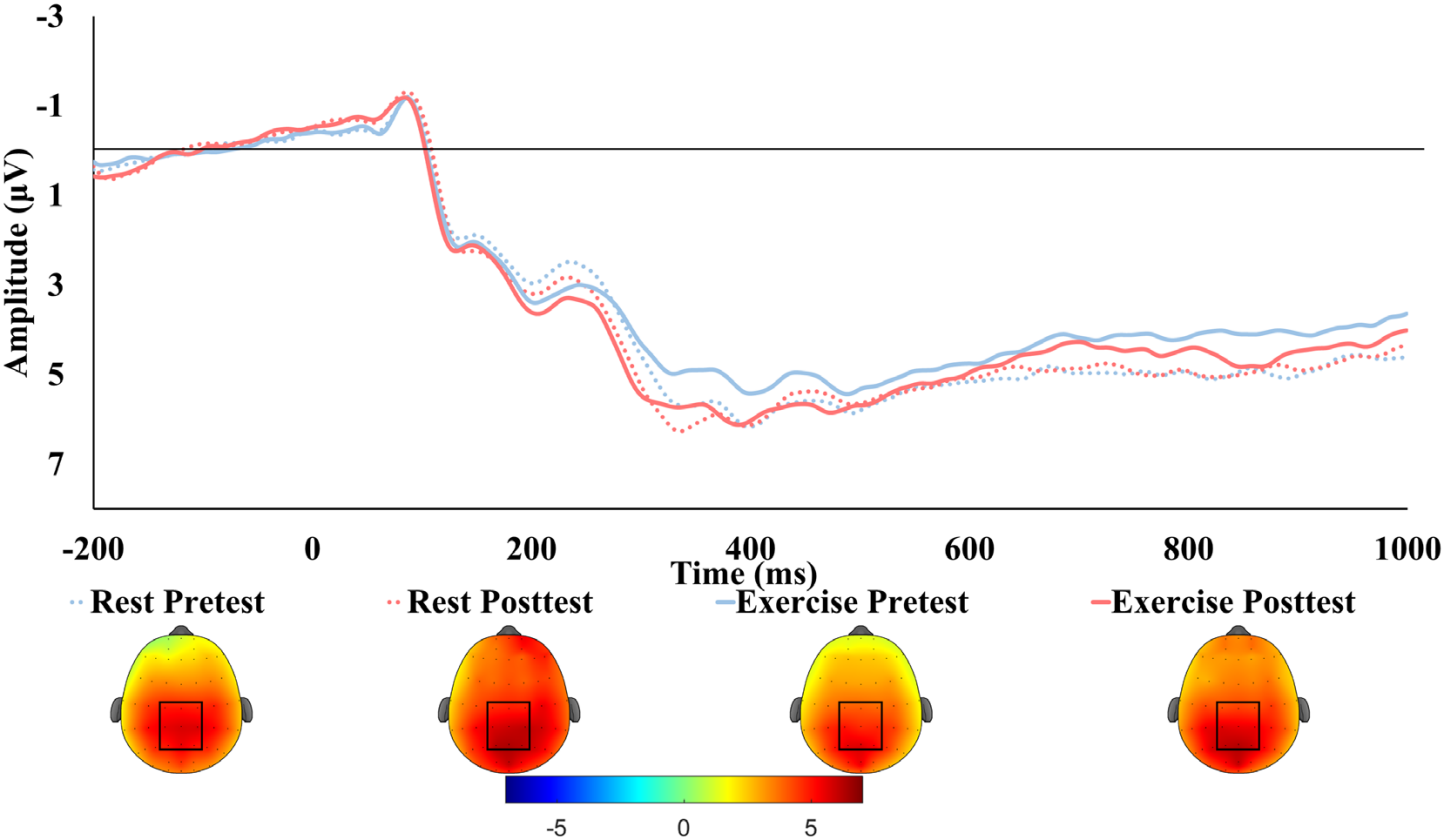
Grand average ERP waveforms during the Nback task averaged across nontarget and target trials. Results showed no difference in P3 amplitude or latency from pretest to posttest for either group. ERP Topoplots averaged across nontarget and target trials, using the mean amplitude from 300 to 500 ms are presented. The ROI used in analysis (CPZ, CP1, CP2, PZ, P1, P2, POZ, PO3, and PO4 electrodes) is designated by a square.

A high number (38–92) and percentage (80%–96%) of trials were retained across both groups, with no significant between‐group difference during any task condition or at any testing timepoint (Table S5). This trial retention exceeds the standard reported in prior literature, which suggests that reliable P3 measures can be achieved with as few as 14–36 usable trials (Cohen and Polich 1997; Duncan et al. 2009; Rietdijk et al. 2014) and N2 amplitude with at least 30 trials (Clayson and Larson 2013). Grand average waveforms (Figures 2 and 3) were constructed using accepted trials, quantified by peak latency and mean amplitude within 50 ms of the peak. The P3 and N2 were based on the largest positive peak within a 300–700 ms post‐stimulus and the largest negative peak within a 200–400 ms post‐stimulus latency window, respectively. These windows were selected according to previous research (Chia‐Liang Tsai et al. 2014; Wen and Tsai 2020) and visual inspection of the grand average ERP waveforms. Regions of interest (ROI) for the P3 (CPZ, CP1, CP2, PZ, P1, P2, POZ PO3, and PO4) and N2 (FZ, F1, F2, FCZ, FC1, and FC2) were defined based on prior literature (Kao et al. 2020), and post hoc topographic images (Figures 2 and 3).

### 2.8 Statistical Methods

Power analyses were conducted a priori using G*Power version 3.1.9.4 (Faul et al. 2007), with *ɑ* = 0.05 and power (1−*β*) = 0.80. Meta‐analyses indicated a large positive effect of acute RE on inhibitory control (SMD = 0.73, Wilke et al. 2019) and a medium positive effect on ERP measures (*g* = 0.47; effect of acute RE on P3 amplitude, Kao et al. 2022). While the primary analysis was a group × time interaction using repeated measures ANOVA, the sample size calculation also considered follow‐up independent‐samples *t*‐tests needed to decompose significant interactions. Thus, based on the most demanding analysis requiring the largest sample, a sample of 114 subjects was needed. To account for potential dropouts, a 10% buffer (11 subjects) was added, increasing the planned sample to 125. A total of 179 subjects were contacted, 125 enrolled, and 123 subjects completed all testing. Subjects were removed for poor task performance (*D‐prime* [hit rate—false alarm rate] ≤ 0; N‐back: *n* = 2) or EEG data (< 20 accepted epochs; Flanker: *n* = 2; N‐back: *n* = 7), resulting in 121 subjects for Flanker analysis and 114 for N‐back analysis. Sensitivity analyses with and without removed subjects did not change the results.

Statistical analyses were conducted using SPSS v.29.0 (IBM; Chicago, IL) with family‐wise alpha set at *p* < 0.05 before Bonferroni correction. Blood pressure and lactate were analyzed using a 2 (Group: RE, rest) × 2 (Time: pre, post) repeated measures ANOVA. An additional Trial Type (congruent/incongruent for the Flanker task; target/nontarget for the N‐back task) within‐subject factor was included for response time, accuracy, and ERP measures.

Exploratory analyses were conducted to examine whether lactate, blood pressure, or HR mediated the effect of group assignment (RE) on cognition for all significant Group × Time interactions. Mediation analyses were conducted for each of the specified mediators using ordinary least squares path analysis (PROCESS v4.1 macro for SPSS; (Hayes 2022)), with 95% confidence intervals for the indirect effect (*a × b*) estimated from 5000 bootstrap samples. To account for individual baseline differences, pretest levels of both cognitive variables and physiological variables (e.g., lactate, blood pressure) were included in each model. Mediation evidence was supported if the confidence interval for the indirect effect did not include zero.

The data that support the findings of this study are available from the corresponding author upon reasonable request.

## 3 Results

Table 2 summarizes physiological and cognitive measures; Table 3 shows significant effects from ANOVA analyses. For brevity, only significant effects involving Group × Time interactions are reported in the text. Additional results are available in the Data S4.

**TABLE 2:** Description of physiological and cognitive measures.

		Exercise		Rest	
		Pretest	Posttest	Pretest	Posttest
Blood (*n* = 121)	Systolic pressure (mmHg)	123.18 (± 1.93)	140.85 (± 2.44)	119.50 (± 1.61)	118.05 (± 1.81)
	Diastolic pressure (mmHg)	75.36 (± 1.39)	73.05 (± 1.22)	70.68 (± 1.31)	71.55 (± 1.27)
	Lactate (mMol/L)	1.38 (± 0.07)	8.22 (± 0.39)	1.37 (± 0.07)	1.91 (± 0.09)
Flanker (*n* = 121)	Congruent accuracy (%)	96.40 (± 0.68)	98.28 (± 0.30)	97.40 (± 0.40)	98.04 (± 0.41)
	Incongruent accuracy (%)	90.59 (± 1.27)	92.90 (±0.78)	92.36 (± 0.74)	93.40 (± 0.74)
	Congruent response time (ms)	371.71 (± 5.90)	352.86 (± 5.19)	379.67 (± 6.02)	372.78 (± 6.41)
	Incongruent response time (ms)	420.35 (± 6.45)	402.00 (± 5.88)	422.16 (± 6.05)	415.80 (± 6.49)
	Congruent P3 amplitude (μV)	8.21 (± 0.52)	7.80 (± 0.53)	7.30 (± 0.52)	7.50 (± 0.52)
	Incongruent P3 amplitude (μV)	8.98 (± 0.50)	8.72 (± 0.57)	7.70 (± 0.54)	7.89 (± 0.53)
	Congruent P3 latency (ms)	393.74 (± 8.50)	367.47 (± 9.44)	389.69 (± 8.30)	394.61 (± 9.99)
	Incongruent P3 latency (ms)	426.71 (± 5.78)	389.76 (± 6.20)	412.80 (± 5.70)	407.39 (± 6.34)
	Congruent N2 amplitude (μV)	−4.17 (± 0.62)	−4.39 (± 0.69)	−4.01 (± 0.79)	−3.89 (± 0.83)
	Incongruent N2 amplitude (μV)	−4.93 (± 0.62)	−4.83 (± 0.70)	−4.07 (± 0.83)	−4.59 (± 0.79)
	Congruent N2 latency (ms)	249.08 (± 4.01)	251.26 (± 4.53)	259.17 (± 5.11)	258.32 (± 5.13)
	Incongruent N2 latency (ms)	252.78 (± 4.23)	250.21 (± 4.20)	257.33 (± 4.17)	257.37 (± 4.37)
Nback (*n* = 114)	Nontarget accuracy (%)	86.88 (± 1.36)	89.39 (± 1.34)	88.49 (± 1.11)	91.01 (± 1.01)
	Target accuracy (%)	82.22 (±1.29)	85.96 (± 1.17)	82.18 (± 1.38)	84.49 (± 1.39)
	Nontarget response time (ms)	760.98 (± 22.47)	683.20 (± 20.37)	771.56 (± 23.49)	732.35 (± 21.23)
	Target response time (ms)	649.18 (± 21.11)	577.89 (± 18.59)	662.59 (± 21.35)	644.36 (± 19.52)
	Nontarget P3 amplitude (μV)	6.78 (± 0.37)	7.35 (± 0.53)	7.29 (± 0.41)	7.52 (± 0.44)
	Target P3 amplitude (μV)	8.41 (± 0.47)	9.33 (± 0.57)	8.85 (± 0.46)	9.17 (± 0.58)
	Nontarget P3 latency (ms)	465.38 (± 14.22)	454.46 (± 13.29)	474.58 (± 13.49)	463.83 (± 12.53)
	Target P3 latency (ms)	422.86 (± 14.02)	415.29 (± 13.22)	430.27 (± 13.54)	426.80 (± 12.61)
	Nontarget N2 amplitude (μV)	−2.85 (± 0.68)	−2.73 (± 0.79)	−2.16 (± 0.80)	−2.56 (± 0.92)
	Target N2 amplitude (μV)	−0.23 (± 0.70)	−0.33 (± 0.73)	−0.07 (± 0.84)	−0.64 (± 0.97)
	Nontarget N2 latency (ms)	265.12 (± 6.04)	262.38 (± 5.35)	254.82 (± 5.15)	256.94 (± 5.26)
	Target N2 latency (ms)	276.00 (± 6.83)	268.53 (± 6.46)	259.05 (± 6.04)	247.21 (± 5.16)

*Note:* Values represent mean ± SE.

**TABLE 3:** Statistical summary of significant repeated measures ANOVA analysis for physiological and cognitive measures.

	Measure	Effect	df	*F*	*p*	ηp^2^
Blood (*n* = 121)	Systolic blood pressure	Group	1, 119	28.23	< 0.01	0.19
		Time	1, 119	65.70	< 0.01	0.36
		Group*Time	1, 119	95.52	< 0.01	0.45
	Diastolic blood pressure	Group	1, 119	7.13	0.01	0.06
		Time	1, 119	25.00	< 0.01	0.17
		Group*Time	1, 119	7.13	0.01	0.06
	Lactate	Group	1, 119	232.58	< 0.01	0.66
		Time	1, 119	316.32	< 0.01	0.73
		Group*Time	1, 119	232.06	< 0.01	0.66
Flanker (*n* = 121)	ACC	Time	1, 119	16.82	< 0.01	0.12
		Congruency	1, 119	148.43	< 0.01	0.56
	RT	Time	1, 119	19.04	< 0.01	0.14
		Congruency	1, 119	619.49	< 0.01	0.84
		Group*Time	1, 119	4.29	0.04	0.04
	P3 ROI amplitude	Congruency	1, 119	16.70	< 0.01	0.12
	P3 ROI latency	Time	1, 119	19.38	< 0.01	0.14
		Congruency	1, 119	36.61	< 0.01	0.22
		Group*Time	1, 119	18.79	< 0.01	0.14
	N2 ROI amplitude	Congruency	1, 119	9.87	< 0.01	0.08
		Group*Time*Congruency	1, 119	4.82	0.03	0.04
Nback (*n* = 114)	ACC	Time	1, 112	28.36	< 0.01	0.20
		Trial Type	1, 112	37.99	< 0.01	0.25
	RT	Time	1, 112	35.95	< 0.01	0.24
		Trial Type	1, 112	280.24	< 0.01	0.71
		Group*Time	1, 112	7.08	0.01	0.06
	P3 ROI amplitude	Trial Type	1, 112	84.14	< 0.01	0.43
	P3 ROI latency	Trial Type	1, 112	40.40	< 0.01	0.27
	N2 ROI amplitude	Trial Type	1, 112	116.23	< 0.01	0.51

*Note:* Effects of all significant ANOVA analyses investigating the effect of acute RE on physiological outcomes, and behavioral and neuroelectric measures of executive function.

### 3.1 Blood Measures

**FIGURE 4:**
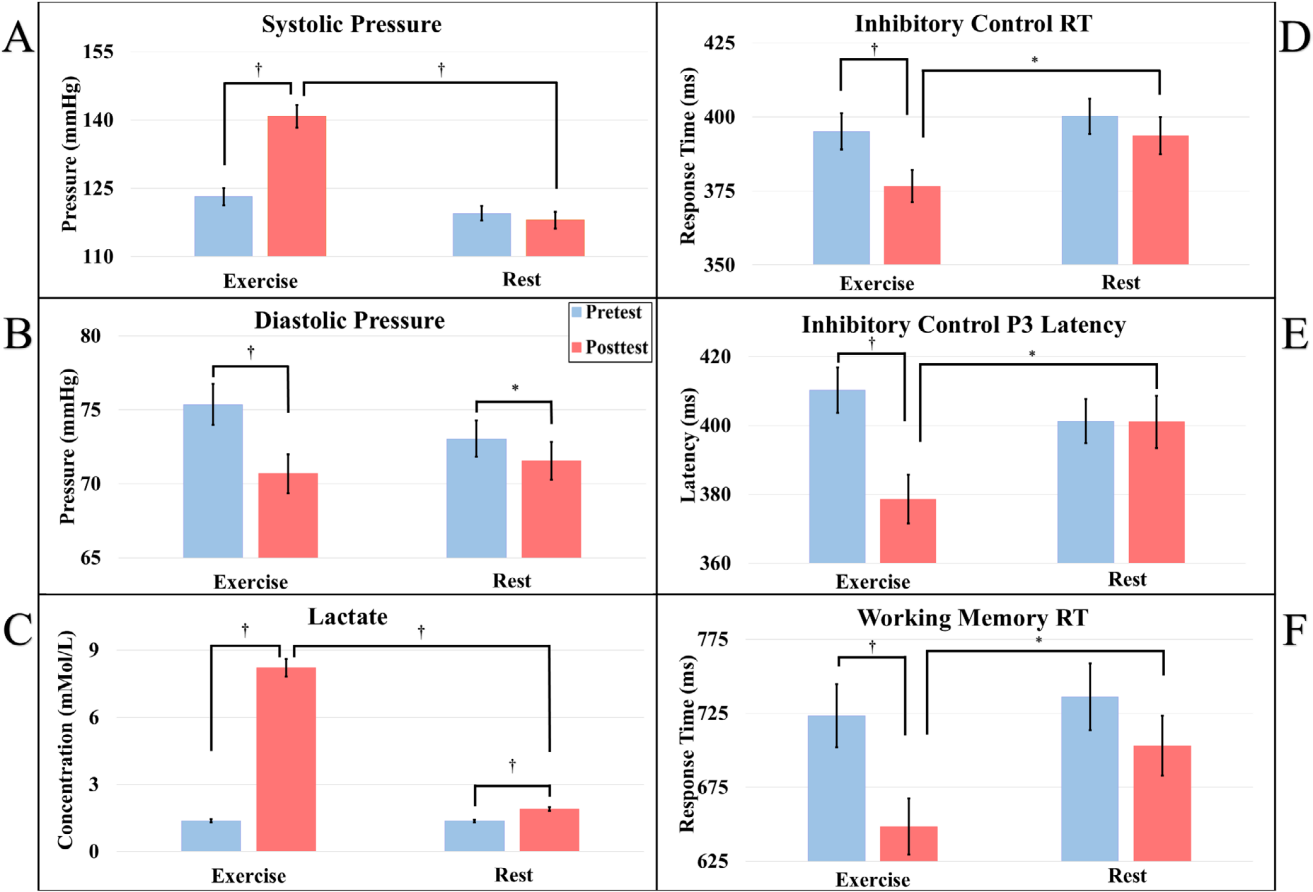
Values represent mean ± SE. Effect of RE or Rest intervention on physiological and cognitive outcomes (* = *p* < 0.05, † = *p* < 0.01) (A) Significant increase in systolic blood pressure after RE but not following rest. (B) Diastolic blood pressure decreased following both RE and rest. (C) Significant increase in lactate concentration following both rest and RE; however, concentration following RE was greater than following rest. (D) While response time (RT) was similar at pretest, RT significantly decreased following RE but not following rest; the RE group had faster RT at posttest than the rest group. (E) P3 latency was similar at pretest but decreased significantly following RE but not rest. P3 latency was shorter for the RE group during the Flanker task than for the rest group at posttest. (F) RT during the N‐back was similar at pretest, but RT decreased only following RE. At posttest, RT was faster for the RE group compared to the rest group.

Among 121 subjects with complete blood pressure and lactate measurements (*n* = 2 blood pressure measurement errors), lactate increased from pretest to posttest for the RE group (*t*_(59)_ = 17.18, *p <* 0.01, *d* = 2.06) and for the rest group (*t*_(60)_ = 4.24, *p <* 0.01, *d* = 0.16). Lactate concentration was higher for the RE group compared to the rest group at posttest (*t*_(119)_ = 15.59, *p <* 0.01, *d* = 1.91) but not at pretest (*t*_(119)_ = 0.38, *p* = 0.35, *d* = 0.01). Systolic blood pressure increased from pretest to posttest for the RE group (*t*_(59)_ = 10.58, *p <* 0.01, *d* = 0.99) but not for the rest group (*t*_(60)_ = 1.37, *p* = 0.08, *d* = 0.09). Systolic pressure was higher at posttest (*t*_(119)_ = 7.73, *p <* 0.01, *d* = 1.41) but not pretest (*t*_(119)_ = 1.66, *p* = 0.05, *d* = 0.23) for the RE group compared to the rest group. Diastolic pressure decreased from pretest to posttest for both the RE (*t*_(59)_ = *−*4.90, *p* < 0.01, *d* = 0.50) and rest group (*t*_(60)_ = *−*1.86, *p* = 0.03, *d* = 0.15), with no differences at pretest (*t*_(119)_ = 1.36, *p* = 0.09, *d* = 0.24) or posttest (*t*_(119)_ = −0.56, *p* = 0.29, *d* = 0.10). Results visualized in Figure 4.

### 3.2 Inhibitory Control

A significant Group × Time interaction on response time and P3 latency was found. The decomposition of these interactions showed that response time (*t*_(60)_ = *−*4.15, *p* < 0.01, *d* = 0.40) and P3 latency (*t*_(60)_ = −5.99, *p <* 0.01, *d* = 0.58) decreased from pretest to posttest for the RE group but not for the rest group (*ts*_(59)_ < 1.75, *ps* > 0.08, *ds* < 0.14). Response time (t_(119)_ = *−*2.05, *p* = 0.02, *d* = 0.37) and P3 latency (*t*_(119)_ = −2.16, *p* = 0.02, *d* = 0.41) were shorter for the RE group compared with the rest group at posttest, but no between‐group difference at pretest (*ts*_(119)_ < 0.98, *ps* > 0.17, *ds* < 0.16), shown in Figure 4. No Group × Time interaction was found for response accuracy or P3 amplitude (*Fs* < 3.20, *ps* > 0.08, *ηp* ^*2*^ *s* < 0.03).

N2 analyses revealed no significant effects involving the Group × Time interactions (*Fs* < *1.73, ps* > *0.19, ηp* ^*2*^ *s < 0.01*). However, decomposition of the significant Group × Time × Congruency interaction showed that in the RE group, N2 amplitude was more negative for the incongruent condition than the congruent condition at pretest (*t*_(60)_ = *6.60, p < 0.01, d* = *0.46*), but this congruency effect was attenuated at posttest (*t*_(60)_ = *1.51, p* = *0.07, d* = *0.19*). In contrast, the rest group showed no difference in N2 amplitude at pretest (*t*_(59)_ = *0.23, p* = *0.41, d* = *0.03*) but at posttest N2 amplitude was more negative in the incongruent compared to the congruent condition (*t*_(59)_ = *2.29, p* = *0.01, d* = *0.30*), visualized in Figure S2.

### 3.3 Working Memory

Decomposition of a significant Group × Time interaction showed that response time decreased from pretest to posttest for the RE group (*t*_(57)_ = −7.01, *p <* 0.01, *d* = 0.46), but not for the rest group (*t*_(55)_ = *−*1.44, *p* = 0.08, *d* = 0.18). There was no between‐group difference at pretest (*t*_(112)_ = −0.41, *p* = 0.34, *d* = 0.01), but response times were shorter for the RE group compared to the rest group at posttest (*t*_(112)_ = −1.99, *p* = 0.02, *d* = 0.48), visualized in Figure 4F. Analysis of accuracy and P3 measures revealed no significant effects involving Group × Time interactions (*Fs* < 1.28, *ps* > 0.26, *ηp* ^*2*^ *s* < 0.01).

### 3.4 Mediation Analyses

**FIGURE 5:**
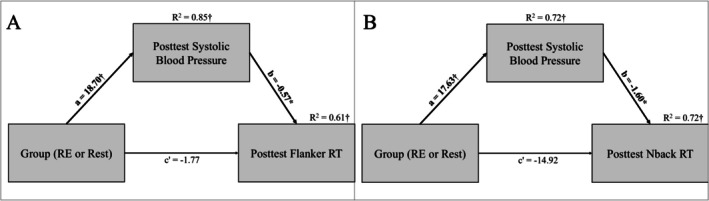
Standardized regression coefficients for the relationship between group assignment and cognitive outcomes as mediated by physiological variables. Results indicate that systolic pressure mediated RE‐induced improvements in response time, identifying systolic blood pressure as a possible mechanism. *R* ^2^ represents the proportion of variance explained by the direct effect (* = *p* < 0.05, † = *p* < 0.01). (A) Systolic blood pressure mediated the relationship between group assignment (RE) and posttest Flanker response time. (B) Systolic blood pressure mediated the relationship between group assignment (RE) and posttest Nback response time.

There was evidence of a direct effect (*a*) of group assignment on systolic pressure (estimate ± SE; 18.70 ± 1.97, *p* < 0.01), and a direct effect (*b*) of posttest systolic pressure on posttest response time (−0.57 ± 0.26, *p* = 0.03). There was no direct effect (c') of group assignment on posttest Flanker response time (−1.77 ± 7.39, *p* = 0.81). As shown in Table 4, an indirect effect (*a × b*) of posttest systolic pressure on posttest Flanker response time (−10.73 ± 4.99, 95% CI = −21.50 to −1.52) indicated higher systolic pressure following RE mediated the improvement in response time. Similarly, after accounting for pretest systolic pressure and N‐back response time, results showed a direct effect (*a*) of group assignment on systolic pressure (17.63 ± 1.89, *p* < 0.01), and a direct effect (*b*) of posttest systolic pressure on posttest response time (−1.60 ± 0.77, *p* = 0.04). No direct effect (c') of group assignment on posttest N‐back response time (−14.92 ± 20.50, *p* = 0.47) was observed. An indirect effect (*a × b*) of posttest systolic pressure on N‐back response time (−28.20 ± 12.38, 95% CI = −53.22 to −5.37) indicated higher systolic pressure following RE mediated the improvement in response time during the N‐back. Results visualized in Figure 5. We did not identify additional evidence in support of other mediations, and neither lactate nor blood pressure mediated the effects on P3 latency. Subsequent sensitivity analyses testing HR as a mediator also did not provide evidence of mediation. Full model coefficients for all the mediation analyses are reported in Tables [Link], [Link], [Link].

**TABLE 4:** Mediation analysis: 95% confidence intervals of the indirect effects (*a × b*) of group assignment on cognitive outcomes through physiological variables.

Indirect effect on posttest cognition	Flanker RT (*n* = 121)				Flanker P3 latency (*n* = 121)				Nback RT (*n* = 114)			
	*a* × *b*	SE	LLCI	ULCI	*a* × *b*	SE	LLCI	ULCI	*a* × *b*	SE	LLCI	ULCI
Posttest systolic pressure	**−10.73**	**4.99**	**−21.37**	**−1.45**	4.41	7.55	−9.48	19.84	**−28.20**	**12.38**	**−54.24**	**−5.70**
Posttest diastolic pressure	1.82	1.39	−0.29	5.23	0.51	1.25	−1.71	3.58	3.78	3.75	−2.74	12.10
Posttest lactate	−0.65	8.42	−21.14	12.86	−1.83	13.93	−28.59	25.40	−14.38	17.40	−48.06	18.70
Posttest heart rate	−3.55	8.12	−20.04	12.24	−13.10	9.77	−32.77	5.43	−23.50	24.07	−71.24	23.94

*Note:* The percentile Bootstrap method (5000 samples) was used to compute the LLCI and ULCI. *a × b* represents the indirect effect of group assignment on posttest cognitive outcomes mediated by the posttest physiological variable. Mediation evidence was supported if the confidence interval for the indirect effect did not include 0, which has been bolded for clarity in the table.

Abbreviations: LLCI, lower limit 95% confidence interval; SE, standard error; ULCI, upper limit 95% confidence interval.

## 4 Discussion

This study examined the effects of acute RE on executive function, exploring physiological mechanisms underlying exercise‐induced cognitive changes. Acute RE reduced response time and P3 latency during inhibitory control, along with decreased working memory response time, suggesting an overall positive effect on executive function processing speed. Furthermore, exploratory analyses indicated that systolic pressure mediated the positive effect of RE on response time during both the Flanker and N‐back tasks. Overall, the findings indicate that acute RE improves behavioral and neuroelectric processing speed during executive functions, potentially through elevated systolic pressure.

Acute RE improved speed but not accuracy during the Flanker task, consistent with a growing body of work showing a positive effect of RE on response time during inhibitory control (Huang et al. 2022; Wilke et al. 2019). Indeed, past reviews report acute exercise largely impacts response time during executive function in adults (Huang et al. 2022; McMorris and Hale 2012); with acute RE in particular showing the greatest effect on inhibitory control (Alves et al. 2014; Brush et al. 2016; Chang et al. 2017; Chang et al. 2014; Chia‐Liang Tsai et al. 2014; Wilke et al. 2019). Despite mixed findings in past studies (Huang et al. 2022; Wilke et al. 2019), our results support an effect of acute RE on working memory. Mixed results in the literature may be due to variability in exercise intensity used, as systematic reviews have shown that only moderate‐intensity RE consistently improves working memory (Huang et al. 2022). Furthermore, moderate‐intensity RE has been shown to improve speed‐related measures during tasks with higher working memory demands (Hsieh et al. 2016), but the impact on measures of accuracy is inconsistent (Brush et al. 2016; Hsieh et al. 2016; Naderi et al. 2019). Altogether, our results add to the growing evidence suggesting that acute moderate‐intensity RE improves processing speed of inhibitory control and working memory, supporting acute RE as a valid approach to enhance executive function.

Faster response times following acute RE were accompanied by changes in neuroelectric measures underlying inhibitory control, specifically decreased P3 latency. These findings differ from past research typically reporting increased P3 amplitude but unchanged P3 latency following acute RE (Kao et al. 2022). This may be due to different cognitive tasks used to elicit P3, as studies using a switching task to assess cognitive flexibility (Wu et al. 2019) and a combined Flanker/Go‐NoGo to assess perceptual/response inhibition (Chia‐Liang Tsai et al. 2014) have found changes in P3 amplitude but not latency. Overall, decreased P3 latency and faster response times in our results suggest that acute RE improves both the behavioral and underlying neuroelectric processing speed of inhibitory control.

Acute RE did not change overall N2 amplitude or latency, consistent with most prior reports (Gusatovic et al. 2022). However, RE appeared to enhance the efficiency of neural processing during conflict resolution. Specifically, at baseline the RE group exhibited an expected interference effect (e.g., greater N2 amplitude for incongruent compared to congruent trials (Fong et al. 2018; Kopp et al. 1996)), which was attenuated following RE. In contrast, the rest group showed the opposite pattern, developing an interference effect after the intervention while one was not present at baseline. This could be interpreted to suggest acute RE enhances neural efficacy during conflict resolution processing, while rest requires a compensatory upregulation of N2 amplitude to maintain task performance. While most studies have not shown an effect of acute exercise on N2 amplitude (Gusatovic et al. 2022), acute AE has occasionally been shown to increase N2 amplitude (Aly and Kojima 2020; Ligeza et al. 2018; Wang et al. 2020; Winneke et al. 2019). The only prior study to include RE (utilizing a combined AE and RE protocol), found decreased N2 amplitude in both the congruent and incongruent trials following exercise, with a larger decrease for the incongruent condition. No change was found in the control condition (Wen and Tsai 2020). Together, these data tentatively suggest acute RE may reduce N2 amplitude, especially incongruent compared to congruent trials, potentially reflecting improved neural efficiency of conflict resolution. However, baseline differences (notably, the absence of an initial interference effect in the rest group) and relatively small change in amplitude following RE confound this interpretation. Therefore, these findings remain preliminary and should be interpreted with caution; more research is needed to determine the acute effect of RE on N2 amplitude.

While subjects' response time improved for inhibitory control and working memory, P3 latency only decreased during inhibitory control. The P3 reflects attention and memory within an information‐processing cascade (Polich 2007), representing the allocation of attentional resources and the updating of mental representations. While inhibitory control is a relatively narrow construct (attention and ability to inhibit prepotent responses (Diamond 2013)), working memory is an expansive, multi‐component, limited‐capacity information processing system (including intertwined attention and memory subsystems (Baddeley 1992, 2003)). Therefore, acute RE may impact attentional processing speed—indexed by P3 latency—affecting the majority of aspects associated with inhibitory control but only a subsection of those associated with working memory. Alternatively, tasks with longer ISI tend to show diminished P3/N2 amplitude and longer latencies (Jeon and Polich 2003; Mathalon and Ford 2002; Zamorano et al. 2014), as was the case in the current N‐back (ISI = 2000 ms) compared to the Flanker (ISI = 1000–1400 ms), potentially decreasing the sensitivity of these measures to detect the acute effects of RE on working memory (Ludyga et al. 2016).

A second objective of this study was to explore physiological markers of RE‐related enhancement in executive function. Our results indicate that increased systolic blood pressure mediated the effect of group assignment (RE) on inhibitory control and working memory response times, whereas lactate, diastolic pressure, and HR did not. Acute exercise, including RE, reduces parasympathetic tone and increases sympathetic nervous system activation during and up to 60 min post exercise (Chiang et al. 2024). This heightened sympathetic activity results in elevated HR and blood pressure (Charkoudian and Rabbitts 2009; Mauss and Robinson 2009), and is linked to arousal (Giuliano et al. 2017). Indeed, acute exercise is thought to enhance cognitive function by increasing arousal (Lambourne and Tomporowski 2010), likely via modulation of the LC‐NE system (McMorris 2016), influencing executive function (McMorris 2016) and attention (Kinomura et al. 1996). While well‐documented following aerobic exercise, NE is also released after RE (Judelson et al. 2008; McMorris 2016). Thus, the beneficial effects of acute RE on cognitive performance may stem from LC‐NE‐mediated arousal. However, since arousal can be indexed by both blood pressure and HR (Critchley et al. 2000; Raz and Lahad 2022), but only systolic blood pressure mediated cognitive effects, these results suggest a distinct role of systolic blood pressure in this process. Future research is needed to clarify the unique role of blood pressure in acute exercise‐induced cognitive improvements.

Lactate has been proposed as a mechanism for acute exercise‐induced improvements in executive function, as it can cross the blood–brain barrier (Liu and Zhou 2024), serve as an alternative energy source to glucose (Liu and Zhou 2024), and contribute to memory processes (Suzuki et al. 2011). Acute exercise increases cortical lactate levels by up to 19% (Dennis et al. 2015; Maddock et al. 2011). Despite a substantial post‐RE increase in lactate, our results indicate that lactate was not a mediator of the observed effects. Prior studies have similarly reported that increased lactate does not correlate with improved executive function (Ando et al. 2022) or object recognition (Baumgartner et al. 2024). However, other studies have reported lactate mediates the positive effects of acute concurrent exercise (a combination of AE and RE) on executive function (Li et al. 2024), but another study from the same group found no mediating effect (Li et al. 2025). Notably, lactate appeared to mediate cognitive flexibility but not inhibitory control in these studies, but neither study found mediation of neuroelectric outcomes. Overall, results suggest lactate increases are not likely to drive acute improvements in behavioral or neuroelectric measures of inhibitory control or working memory, though its role in cognitive flexibility may warrant further investigation.

Limitations of this study include substantial individual variability in lactate response to RE (range: 2.8–18.3 mMol/L), exceeding expectations for moderate‐intensity exercise. This may reflect individual differences in lactate production/clearance (Brown et al. 1990; Jones et al. 2015; Stallknecht et al. 1998), or factors like submaximal effort or 1RM estimation errors (Richens and Cleather 2014). The fixed ISI during the N‐back task may have impacted ERP components through anticipatory effects, though task complexity may have mitigated substantial confounding. Additionally, HR remained elevated only in the RE group during posttest cognitive testing (Figure S3), potentially influencing executive function outcomes. However, HR was allowed to return to within 10% of baseline, consistent with prior studies investigating the acute effect of exercise on cognition (Hillman et al. 2003; Nanda et al. 2013; Tsai et al. 2018), and did not mediate the effect.

Strengths include being among the first empirical studies investigating the acute effects of RE on executive function, and one of the first to explore blood‐based physiological markers following acute RE, with the aim of identifying potential mechanisms in the relationship for future research. The study was well‐powered and used a standardized 1RM protocol to ensure a personalized and repeatable exercise intensity. Finally, the sex‐matched control video condition helped isolate the effects of physical exertion by maintaining cognitive engagement and emotional responses similar to RE, reducing confounds associated with traditional seated reading controls. While no control condition is without trade‐offs, this design balanced comparability and feasibility.

## 5 Conclusions

This study contributes to our understanding of the relationship between acute RE and cognitive function. Results demonstrate that acute RE positively impacts executive functions, enhancing processing speed during inhibitory control and working memory and improving neuroelectric function during inhibitory control. Such enhanced behavioral processing response speeds may be mediated by RE‐induced increases in systolic blood pressure, although more research is needed to confirm this finding. Overall, the current findings highlight the potential of acute RE as an effective strategy to enhance executive function. Clinically, these findings support the integration of RE into exercise programs for acute benefits to executive functions.

## Author Contributions

**Nicholas W. Baumgartner:** conceptualization, data curation, formal analysis, investigation, methodology, project administration, visualization, writing – original draft, writing – review and editing. **Michael D. Belbis:** data curation, investigation, methodology, writing – review and editing. **Kyoungmin Noh:** project administration, writing – review and editing. **Daniel M. Hirai:** conceptualization, methodology, resources, supervision, validation, writing – review and editing. **Steve Amireault:** conceptualization, formal analysis, methodology, supervision, validation, writing – review and editing. **Shih‐Chun Kao:** conceptualization, data curation, formal analysis, investigation, methodology, project administration, resources, software, supervision, validation, visualization, writing – original draft, writing – review and editing.

## Conflicts of Interest

The authors declare no conflicts of interest.

## Supporting information


**Figure S1:** Example tasks measuring executive function. (A) Modified Eriksen Flanker Task of inhibitory control. Both congruent and incongruent conditions were presented for 200 ms, with equiprobable jittered intertrial intervals of 1000, 1200, or 1400 ms. (B) Serial N‐back task of working memory. Both target and nontarget conditions were presented for 200 ms, with a fixed inter‐stimulus interval of 2300 ms. Subjects were presented with a series of colored shapes one at a time and instructed to press the “Red” button if the shape currently presented was the same as the shape presented two shapes ago (target trials), and the “blue” button if the present shape was different than the shape presented two shapes ago (nontarget trials).

**Figure S2:** Effects of acute RE on N2 amplitude for each Flanker congruency condition (* = *p* < 0.05, † = *p* < 0.01) (A) Grand average ERP waveforms for the RE (left) and rest (right) groups during the Flanker task, separately for congruent and incongruent trials across the ROI (FZ, F1, F2, FCZ, FC1, and FC2 electrodes). (B) At pretest, N2 amplitude was more negative for the incongruent compared to the congruent trials in the RE group, but there was no difference in the rest group. (C) At posttest, N2 amplitude was more negative for the incongruent compared to the congruent trials for the rest group, but there was no longer a difference for the RE group.

**Figure S3:** Sensitivity analyses involving heart rate (HR) during and following the intervention, values represent mean ± SE (* = *p* < 0.05, † = *p* < 0.01). (A) HR increased following RE, while there was no change following rest. (B) HR across testing on Day 2, error bars represent ± SE. The gray box denotes HR during the intervention, taken at the same time across both interventions. HR at pretest was not different between groups, but during the intervention the RE group had significantly higher mean HR. After the intervention, HR for the RE group decreased but remained elevated compared to the rest group.

**Data S1:** psyp70171‐sup‐0004‐DataS1.docx.

**Table S1:** Accepted trials.

**Table S2:** Flanker response time mediation analyses.

**Table S3:** Flanker P3 latency mediation analyses.

**Table S4:** N‐back response time mediation analyses.
