# Supplementary figures and images for: Brawn and Brainpower: Acute Resistance Exercise Improves Behavioral and Neuroelectric Measures of Executive Function
Source: Psychophysiology. 2025 Oct 30;62(11):e70171. doi: 10.1111/psyp.70171 (PMC12575885; doi:10.1111/psyp.70171)

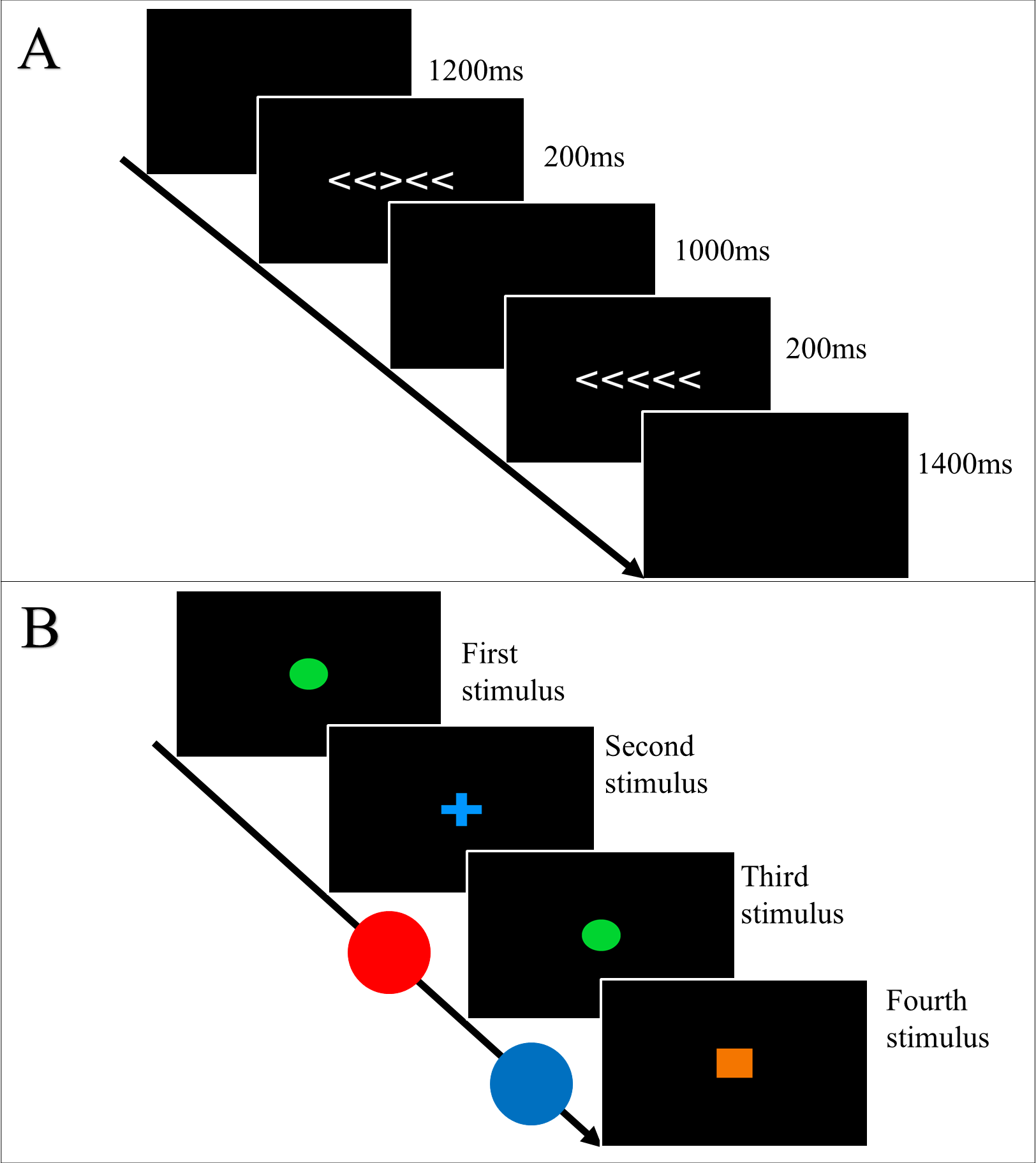

Supplement: Supplementary file 1 — Figure S1: Example tasks measuring executive function. (A) Modified Eriksen Flanker Task of inhibitory control. Both congruent and incongruent conditions were presented for 200 ms, with equiprobable jittered intertrial intervals of 1000, 1200, or 1400 ms. (B) Serial N‐back task of working memory. Both target and nontarget conditions were presented for 200 ms, with a fixed inter‐stimulus interval of 2300 ms. Subjects were presented with a series of colored shapes one at a time and instructed to press the “Red” button if the shape currently presented was the same as the shape presented two shapes ago (target trials), and the “blue” button if the present shape was different than the shape presented two shapes ago (nontarget trials). [file PSYP-62-e70171-s002.tif]

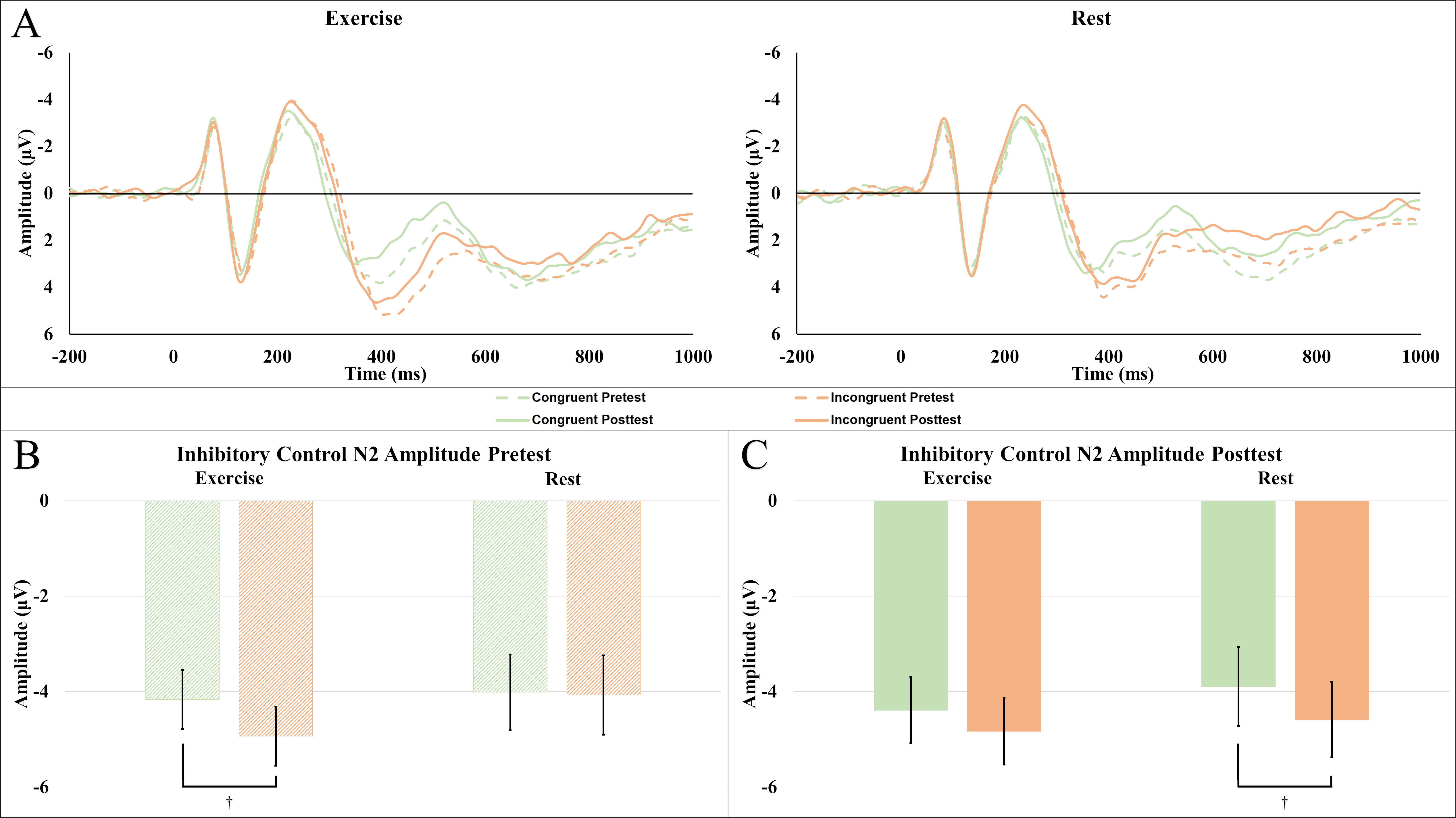

Supplement: Supplementary file 2 — Figure S2: Effects of acute RE on N2 amplitude for each Flanker congruency condition (* = p < 0.05, † = p < 0.01) (A) Grand average ERP waveforms for the RE (left) and rest (right) groups during the Flanker task, separately for congruent and incongruent trials across the ROI (FZ, F1, F2, FCZ, FC1, and FC2 electrodes). (B) At pretest, N2 amplitude was more negative for the incongruent compared to the congruent trials in the RE group, but there was no difference in the rest group. (C) At posttest, N2 amplitude was more negative for the incongruent compared to the congruent trials for the rest group, but there was no longer a difference for the RE group. [file PSYP-62-e70171-s003.tif]

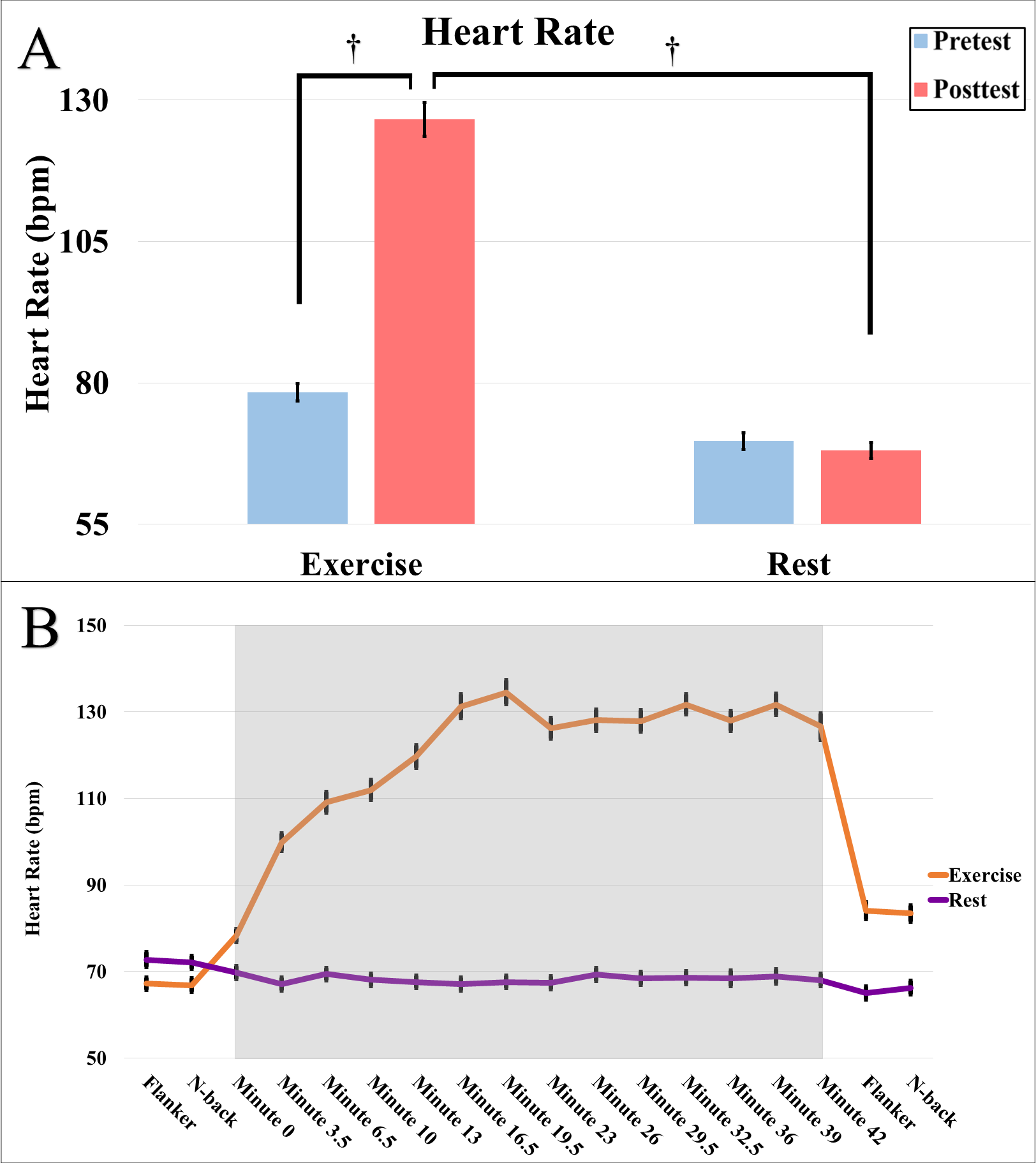

Supplement: Supplementary file 3 — Figure S3: Sensitivity analyses involving heart rate (HR) during and following the intervention, values represent mean ± SE (* = p < 0.05, † = p < 0.01). (A) HR increased following RE, while there was no change following rest. (B) HR across testing on Day 2, error bars represent ± SE. The gray box denotes HR during the intervention, taken at the same time across both interventions. HR at pretest was not different between groups, but during the intervention the RE group had significantly higher mean HR. After the intervention, HR for the RE group decreased but remained elevated compared to the rest group. [file PSYP-62-e70171-s005.tif]
