# Supplementary material for: Brawn and Brainpower: Acute Resistance Exercise Improves Behavioral and Neuroelectric Measures of Executive Function
Source: Psychophysiology. 2025 Oct 30;62(11):e70171. doi: 10.1111/psyp.70171 (PMC12575885; doi:10.1111/psyp.70171)
Supplement: Supplementary file 4 — Data S1: psyp70171‐sup‐0004‐DataS1.docx. [file PSYP-62-e70171-s004.docx]

**S.1 Aerobic Fitness**

Maximum oxygen consumption (V̇O_2max_) was assessed using a computerized indirect calorimetry system (COSMED, Quark CPET OMNIA; Concord, CA) that measured oxygen uptake (V̇O_2_) and respiratory exchange ratio (RER) on a breath-by-breath basis. A modified Balke protocol (Balke & Ware, 1959) was performed on a motorized treadmill (Trackmaster, TMX428; Lexington, KY) at a constant speed with a 2% increase in grade every two minutes until volitional exhaustion. Heart rate (HR) was continuously measured with a HR monitor (Garmin, HRM-Dual; Olathe, KS), and ratings of perceived exertion (RPE) (Borg, 1982) and Feeling Scale (Hardy & Rejeski, 1989) were recorded every two minutes. Height and weight were measured using a stadiometer (Seca, model 240; Hamburg, Germany) and digital scale (Tanita, WB-300 Plus; Tokyo, Japan). Relative V̇O_2max_ was expressed in ml/kg/min, sampled breath-by-breath, expressed as a 15-breath moving average. Maximal effort was confirmed by a plateau in oxygen uptake (>2 ml/kg/min despite increased workload) or at least 2 of the following criteria: 1) a peak HR ≥ 90% age-predicted maximum HR (HRmax); 2) RER ≥ 1.10; and/or 3) RPE ≥ 17.

**S.2 Results**

**S2.1 Blood Measures**

Analysis of blood lactate revealed time and group main effects, with higher blood lactate levels at posttest (Mean ± SE; 5.07 mMol/L ± 0.20) and in the RE group (4.82 mMol/L ± 0.15) compared to at pretest (1.38 mMol/L ± 0.05) and in the rest group (1.63 mMol/L ± 0.15). Analysis of systolic blood pressure revealed time and group main effects, with higher systolic blood pressure at posttest (129.49 mmHg ± 1.50) and in the RE group (132.37 mmHg ± 1.82) compared to at pretest (121.60 mmHg ± 1.23) and in the rest group (118.73 mmHg ± 1.81). Analysis of diastolic blood pressure revealed Time and group main effects, with lower diastolic blood pressure at posttest (71.04 mmHg ±0.90) and in the rest group (72.30 mmHg ± 1.19) compared to at pretest (74.31 mmHg ± 0.91) and in the RE group (73.04 mmHg ± 1.20).

**S2.2 Inhibitory Control**

Analysis of accuracy revealed time and congruency main effects, with higher scores at posttest (95.68% ± 0.36) and for congruent trials (97.55% ± 0.29) than at pretest (94.19% ± 0.53) and incongruent trials (92.31% ± 0.60). Analysis of response time showed time and congruency main effects, with shorter response times at posttest (385.86 ms ± 4.13) and for congruent trials (369.26 ms ± 3.89) than for pretest (398.37 ms ± 4.20) and incongruent trials (415.08 ms ± 4.15). Analysis of P3 amplitude revealed a congruency main effect, with higher P3 amplitude for incongruent (8.32 μV ± 0.35) than congruent trials (7.70 μV ± 0.34). Analysis of P3 latency showed time and congruency main effects, with shorter P3 latency at posttest (389.81 ms ± 5.17) and for congruent trials (386.38 ms ± 5.87) than pretest (405.74 ms ± 4.59) and incongruent trials (409.17 ms ± 3.85). Analysis of N2 amplitude revealed a congruency main effect, with more negative N2 amplitude for incongruent (-4.60 *μV ±0.50*) than congruent trials (-4.11 *μV ± 0.49*).

**S2.3 Working Memory**

Analysis on accuracy revealed Time and Trial Type main effects, with higher scores at posttest (87.71% ± 0.75) and for nontarget trials (88.94% ± 0.83) than at pretest (84.94% ± 0.78) and target trials (83.71% ± 0.84). Analysis of response time showed Time and Trial Type main effects, with shorter response times at posttest (659.45 ms ± 13.61) and for nontarget trials (633.51 ms ± 13.48) compared to pretest (711.08 ms ± 15.21) and target trials (737.03 ms ± 14.73). Analysis of P3 amplitude revealed a Trial Type main effect, with higher P3 amplitude for target (8.94 μV ± 0.34) compared to nontarget trials (7.23 μV ± 0.28). Analysis of P3 latency showed a Trial Type main effect, with shorter P3 latency for target trials (423.81 ms ± 8.13) compared to nontarget (464.65ms ± 8.56). Analysis of N2 amplitude revealed a Trial Type main effect, with more negative N2 amplitude for nontarget (-2.58 *μV ± 0.55*) compared to target (-0.28 *μV ± 0.55*).

**References**

Balke, B., & Ware, R. (1959). An experimental study of physical fitness of Air Force personnel. *U S Armed Forces Med J* , *10*(6), 675–688.

Borg, G. A. V. (1982). Psychophysical bases of perceived exertion. *Medicine & Science in Sports & Exercise*, *14*(5), 377–381.

Hardy, C. J., & Rejeski, W. J. (1989). Not What, but How One Feels: The Measurement of Affect during Exercise. *Journal of Sport and Exercise Psychology*, *11*(3), 304–317. https://doi.org/10.1123/JSEP.11.3.304
