# Supplementary material for: Brawn and Brainpower: Acute Resistance Exercise Improves Behavioral and Neuroelectric Measures of Executive Function
Source: Psychophysiology. 2025 Oct 30;62(11):e70171. doi: 10.1111/psyp.70171 (PMC12575885; doi:10.1111/psyp.70171)
Supplement: Supplementary file 5 — Table S1: Accepted trials. [file PSYP-62-e70171-s008.docx]

| **Table S1. Accepted Trials** | | | | | | |
| --- | --- | --- | --- | --- | --- | --- |
|  | **Total** | **RE Group** | | **Rest Group** | | **Group Difference** |
|  |  | mean ± SD | % | mean ± SD | % | p-value |
| Flanker | n = 121 | n = 62 | | n = 59 | |  |
| Congruent pretest | 90.80 ± 8.10 | 91.03 ± 6.03 | 94.8% | 90.57 ± 9.82 | 94.3% | 0.75 |
| Incongruent pretest | 85.88 ± 10.15 | 85.52 ± 10.38 | 89.1% | 86.25 ± 9.98 | 89.8% | 0.70 |
| Congruent posttest | 91.41 ± 9.37 | 90.07 ± 11.87 | 93.8% | 92.78 ± 5.60 | 96.6% | 0.11 |
| Incongruent posttest | 86.24 ± 10.33 | 84.72 ± 12.22 | 88.3% | 87.78 ± 7.77 | 91.4% | 0.10 |
| Nback | n = 114 | n = 57 | | n = 57 | |  |
| Nontarget pretest | 78.96 ± 9.29 | 78.47 ± 10.26 | 85.3% | 79.46 ± 8.16 | 86.4% | 0.42 |
| Target pretest | 38.45 ± 5.22 | 38.43 ± 5.16 | 80.1% | 38.46 ± 5.33 | 80.1% | 0.98 |
| Nontarget posttest | 80.93 ± 9.56 | 80.00 ± 10.16 | 87.0% | 81.89 ± 8.88 | 89.0% | 0.11 |
| Target posttest | 39.83 ± 5.23 | 40.05 ± 4.96 | 83.4% | 39.61 ± 5.60 | 82.5% | 0.81 |
| Number of trials accepted for each task at pretest and posttest by intervention. Group differences were tested with an independent samples t-test for mean difference between RE and rest group. | | | | | | |
