# Supplementary material for: Brawn and Brainpower: Acute Resistance Exercise Improves Behavioral and Neuroelectric Measures of Executive Function
Source: Psychophysiology. 2025 Oct 30;62(11):e70171. doi: 10.1111/psyp.70171 (PMC12575885; doi:10.1111/psyp.70171)
Supplement: Supplementary file 6 — Table S2: Flanker response time mediation analyses. [file PSYP-62-e70171-s007.docx]

| **Table S2. Flanker Response Time Mediation Analyses** | | | | | | | | | | |  |  |
| --- | --- | --- | --- | --- | --- | --- | --- | --- | --- | --- | --- | --- |
| *n* = 121 | Outcome Variable | | | | | | | | | |  |  |
|  | Posttest Systolic Blood Pressure | | | | | Posttest Flanker Response Time | | | | |  |  |
|  |  |  |  |  |  |  |  |  |  |  |  | |
| Variable | Coeff. | *SE* | *p* | *LLCI* | *ULCI* | Coeff. | *SE* | *p* | LLCI | ULCI |  |  |
| Group | **18.70** | **1.97** | **< 0.01** | **14.81** | **22.60** | -1.77 | 7.39 | 0.81 | -16.40 | 12.87 |  |  |
| Posttest Systolic | – | – | – | – | – | **-0.57** | **0.26** | **0.03** | **-1.09** | **-0.05** |  |  |
| Pretest Systolic | **0.94** | **0.07** | **< 0.01** | **0.80** | **1.08** | 0.53 | 0.53 | 0.10 | -0.10 | 1.16 |  |  |
| Pretest Flanker RT | 0.00 | 0.02 | 0.95 | -0.04 | 0.04 | **0.75** | **0.06** | **< 0.01** | **0.63** | **0.87** |  |  |
| Constant | 5.19 | 12.01 | 0.67 | -18.60 | 28.98 | **98.67** | **33.80** | **< 0.01** | **31.71** | **165.62** |  |  |
|  | *R*^2^ = 0.85 | | |  |  | *R*^2^ = 0.61 | | |  |  |  |  |
|  | *F*(3,117) = 103.66, *p* <0.01 | | | | | *F*(4,116) = 43.77, *p* < 0.01 | | | | |  | |
| Indirect effect of Group on Posttest Flanker RT | | | | | | | | | | |  |  |
|  | *Effect* | *SE* |  | *LLCI* | *ULCI* |  |  |  |  |  |  |  |
| Posttest Systolic | **-10.73** | **4.99** |  | **-21.50** | **-1.52** |  |  |  |  |  |  |  |
| *n* = 121 | Outcome Variable | | | | | | | | | |  | |
|  | Posttest Diastolic Blood Pressure | | | | | Posttest Flanker Response Time | | | | |  | |
|  |  |  |  |  |  |  |  |  |  |  |  | |
| Variable | Coeff. | *SE* | *p* | *LLCI* | *ULCI* | Coeff. | *SE* | *p* | LLCI | ULCI |  |  |
| Group | **-2.87** | **1.25** | **0.02** | **-5.35** | **-0.39** | **-14.52** | **5.69** | **0.01** | **-25.78** | **-3.25** |  |  |
| Posttest Diastolic | – | – | – | – | – | -0.63 | 0.41 | 0.13 | -1.46 | 0.19 |  |  |
| Pretest Diastolic | **0.74** | **0.06** | **< 0.01** | **0.61** | **0.86** | 0.52 | 0.41 | 0.21 | -0.30 | 1.34 |  |  |
| Pretest Flanker RT | 0.01 | 0.01 | 0.52 | -0.02 | 0.04 | **0.75** | **0.06** | **< 0.01** | **0.63** | **0.87** |  |  |
| Constant | 14.27 | 7.43 | 0.06 | -0.45 | 28.99 | **98.96** | **33.60** | **< 0.01** | **32.40** | **165.52** |  |  |
|  | *R*^2^ = 0.55 | | |  |  | *R*^2^ = 0.60 | | |  |  |  |  |
|  | *F*(3,117) = 46.47, *p* <0.01 | | | | | *F*(4,116) = 42.30, *p* < 0.01 | | | | |  | |
| Indirect effect of Group on Posttest Flanker RT | | | | | | | | | | |  |  |
|  | *Effect* | *SE* |  | *LLCI* | *ULCI* |  |  |  |  |  |  |  |
| Posttest DBP | 1.82 | 1.39 |  | -0.20 | 5.12 |  |  |  |  |  |  |  |
| *n* = 121 | Outcome Variable | | | | | | | | | |  | |
|  | Posttest Lactate | | | | | Posttest Flanker Response Time | | | | |  | |
|  |  |  |  |  |  |  |  |  |  |  |  | |
| Variable | Coeff. | *SE* | *p* | *LLCI* | *ULCI* | Coeff. | *SE* | *p* | LLCI | ULCI |  |  |
| Group | **6.28** | **0.40** | **< 0.01** | **5.48** | **7.08** | -12.50 | 9.62 | 0.20 | -31.55 | 6.56 |  |  |
| Posttest Lactate | – | – | – | – | – | -0.10 | 1.26 | 0.93 | -2.60 | 2.39 |  |  |
| Pretest Lactate | 0.10 | 0.36 | 0.79 | -0.62 | 0.82 | -2.42 | 4.95 | 0.63 | -12.23 | 7.38 |  |  |
| Pretest Flanker RT | -0.01 | 0.00 | 0.13 | -0.02 | 0.00 | **0.75** | **0.06** | **< 0.01** | **0.63** | **0.87** |  |  |
| Constant | **4.40** | **1.83** | **0.02** | **0.78** | **8.03** | **98.84** | **25.51** | **< 0.01** | **48.31** | **149.37** |  |  |
|  | *R*^2^ = 0.68 | | |  |  | *R*^2^ = 0.59 | | |  |  |  |  |
|  | *F*(3,117) = 82.85, *p* <0.01 | | | | | *F*(4,116) = 41.79, *p* < 0.01 | | | | |  | |
| Indirect effect of Group on Posttest Flanker RT | | | | | | | | | | |  | |
|  | *Effect* | *SE* |  | *LLCI* | *ULCI* |  |  |  |  |  |  |  |
| Posttest Lactate | -0.65 | 8.42 |  | -21.14 | 12.35 |  |  |  |  |  |  |  |
| *n* = 121 | Outcome Variable | | | | | | | | | |  | |
|  | Posttest Heart Rate | | | | | Posttest Flanker Response Time | | | | |  | |
|  |  |  |  |  |  |  |  |  |  |  |  | |
| Variable | Coeff. | *SE* | *p* | *LLCI* | *ULCI* | Coeff. | *SE* | *p* | LLCI | ULCI |  |  |
| Group | **54.15** | **3.51** | **< 0.01** | **47.20** | **61.11** | -11.10 | 10.28 | 0.28 | -31.46 | 9.26 |  |  |
| Posttest HR | – | – | – | – | – | -0.07 | 0.16 | 0.67 | -0.37 | 0.24 |  |  |
| Pretest HR | **0.50** | **0.14** | **< 0.01** | **0.22** | **0.78** | 0.22 | 0.25 | 0.39 | -0.28 | 0.71 |  |  |
| Pretest Flanker RT | 0.00 | 0.01 | 0.99 | -0.07 | 0.07 | **0.75** | **0.06** | **< 0.01** | **0.63** | **0.87** |  |  |
| Constant | 33.06 | 17.98 | 0.07 | -2.56 | 68.67 | **84.14** | **30.58** | **0.01** | **23.57** | **144.71** |  |  |
|  | *R*^2^ = 0.74 | | |  |  | *R*^2^ = 0.59 | | |  |  |  |  |
|  | *F*(3,117) = 109.83, *p* <0.01 | | | | | *F*(4,116) = 41.09, *p* < 0.01 | | | | |  | |
| Indirect effect of Group on Posttest Flanker RT | | | | | | | | | | |  | |
|  | *Effect* | *SE* |  | *LLCI* | *ULCI* |  |  |  |  |  |  |  |
| Posttest HR | -3.55 | 8.12 |  | -20.04 | 12.24 |  |  |  |  |  |  |  |
| Results of regression analysis accounting for pretest Flanker response time and physiological variables, modeling posttest physiological variable (for estimating *a*) and posttest Flanker response time (for estimating *b*). Significant direct and indirect (*a × b*) effects are **bolded** for clarity. RT = response time, HR = heart rate, SE = standard error. Lower limit (LLCI) and upper limit (ULCI) 95% confidence intervals were calculated based on 5,000 bootstrap samples. | | | | | | | | | | |  |  |
