# Supplementary material for: Brawn and Brainpower: Acute Resistance Exercise Improves Behavioral and Neuroelectric Measures of Executive Function
Source: Psychophysiology. 2025 Oct 30;62(11):e70171. doi: 10.1111/psyp.70171 (PMC12575885; doi:10.1111/psyp.70171)
Supplement: Supplementary file 7 — Table S3: Flanker P3 latency mediation analyses. [file PSYP-62-e70171-s001.docx]

| **Table S3. Flanker P3 Latency Mediation Analyses** | | | | | | | | | | | | |  |  |
| --- | --- | --- | --- | --- | --- | --- | --- | --- | --- | --- | --- | --- | --- | --- |
| *n* = 121 | Outcome Variable | | | | | | | | | | | |  |  |
|  | Posttest Systolic Blood Pressure | | | | | | Posttest Flanker P3 Latency | | | | | |  |  |
|  |  |  |  |  |  |  |  |  |  |  |  |  |  | |
| Variable | Coeff. | *SE* | *p* | *LLCI* | *ULCI* | Coeff. | | *SE* | *p* | LLCI | ULCI |  | |  |
| Group | **19.01** | **1.92** | **< 0.01** | **15.20** | **22.83** | **-33.11** | | **9.92** | **< 0.01** | **-52.77** | **-13.45** |  | |  |
| Posttest Systolic | – | – | – | – | – | 0.23 | | 0.35 | 0.51 | -0.47 | 0.93 |  | |  |
| Pretest Systolic | **0.95** | **0.07** | **< 0.01** | **0.81** | **1.09** | -0.59 | | 0.43 | 0.17 | 0.17 | 0.26 |  | |  |
| Pretest P3 Latency | **-0.04** | **0.02** | **0.03** | **-0.08** | **-0.01** | **0.84** | | **0.07** | **< 0.01** | **0.69** | **0.98** |  | |  |
| Constant | 21.77 | 11.05 | 0.05 | -0.12 | 43.67 | **107.49** | | **42.61** | **0.01** | **23.07** | **191.91** |  | |  |
|  | *R*^2^ = 0.74 | | |  |  | *R*^2^ = 0.56 | | | |  |  |  | |  |
|  | *F*(3,117) = 109.96, *p* <0.01 | | | | | | *F*(4,116) = 36.52, *p* < 0.01 | | | | | |  | |
| Indirect effect of Group on Posttest Flanker P3 Latency | | | | | | | | | | | | |  | |
|  | *Effect* | *SE* |  | *LLCI* | *ULCI* |  | |  |  |  |  |  | |  |
| Posttest Systolic | 4.41 | 7.55 |  | -9.30 | 20.25 |  | |  |  |  |  |  | |  |
| *n* = 121 | Outcome Variable | | | | | | | | | | | |  | |
|  | Posttest Diastolic Blood Pressure | | | | | | Posttest Flanker P3 Latency | | | | | |  | |
|  |  |  |  |  |  |  |  |  |  |  |  |  |  | |
| Variable | Coeff. | *SE* | *p* | *LLCI* | *ULCI* | Coeff. | | *SE* | *p* | LLCI | ULCI |  | |  |
| Group | **-2.65** | **1.23** | **0.03** | **-5.08** | **-0.22** | **-29.55** | | **7.46** | **< 0.01** | **-44.32** | **-14.78** |  | |  |
| Posttest Diastolic | – | – | – | – | – | -0.19 | | 0.56 | 0.73 | -1.29 | 0.91 |  | |  |
| Pretest Diastolic | **0.73** | **0.06** | **< 0.01** | **0.61** | **0.85** | -0.29 | | 0.54 | 0.59 | -1.37 | 0.78 |  | |  |
| Pretest P3 Latency | **-0.03** | **0.01** | **0.02** | **-0.05** | **-0.01** | **0.81** | | **0.07** | **< 0.01** | **0.67** | **0.96** |  | |  |
| Constant | **29.88** | **6.77** | **< 0.01** | **16.47** | **43.29** | **109.33** | | **43.63** | **0.01** | **22.91** | **195.75** |  | |  |
|  | *R*^2^ = 0.57 | | |  |  | *R*^2^ = 0.56 | | | |  |  |  | |  |
|  | *F*(3,117) = 50.21, *p* <0.01 | | | | | | *F*(4,116) = 36.04, *p* < 0.01 | | | | | |  | |
| Indirect effect of Group on Posttest Flanker P3 Latency | | | | | | | | | | | | |  | |
|  | *Effect* | *SE* |  | *LLCI* | *ULCI* |  | |  |  |  |  |  | |  |
| Posttest Diastolic | 0.51 | 1.25 |  | -1.60 | 3.56 |  | |  |  |  |  |  | |  |
| *n* = 121 | Outcome Variable | | | | | | | | | | | |  | |
|  | Posttest Lactate | | | | | | Posttest Flanker P3 Latency | | | | | |  | |
|  |  |  |  |  |  |  |  |  |  |  |  |  |  | |
| Variable | Coeff. | *SE* | *p* | *LLCI* | *ULCI* | Coeff. | | *SE* | *p* | LLCI | ULCI |  | |  |
| Group | **6.37** | **0.40** | **< 0.01** | **5.57** | **7.17** | **-27.95** | | **12.67** | **0.03** | **-53.05** | **-2.85** |  | |  |
| Posttest Lactate | – | – | – | – | – | -0.29 | | 1.64 | 0.86 | -3.54 | 2.96 |  | |  |
| Pretest Lactate | 0.08 | 0.36 | 0.83 | -0.64 | 0.80 | -1.78 | | 6.45 | 0.78 | -14.56 | 11.00 |  | |  |
| Pretest P3 Latency | -0.01 | 0.00 | 0.14 | -0.01 | 0.00 | **0.82** | | **0.07** | **< 0.01** | **0.68** | **0.97** |  | |  |
| Constant | **4.19** | **1.72** | **0.02** | **0.79** | **7.59** | **73.70** | | **31.23** | **0.02** | **11.85** | **135.55** |  | |  |
|  | *R*^2^ = 0.68 | | |  |  | *R*^2^ = 0.55 | | | |  |  |  | |  |
|  | *F*(3,117) = 82.78, *p* <0.01 | | | | | | *F*(4,116) = 35.88, *p* < 0.01 | | | | | |  | |
| Indirect effect of Group on Posttest Flanker P3 Latency | | | | | | | | | | | | |  | |
|  | *Effect* | *SE* |  | *LLCI* | *ULCI* |  | |  |  |  |  |  | |  |
| Posttest Lactate | -1.83 | 13.93 |  | -28.14 | 25.66 |  | |  |  |  |  |  | |  |
| *n* = 121 | Outcome Variable | | | | | | | | | | | |  | |
|  | Posttest Heart Rate | | | | | | Posttest Flanker P3 Latency | | | | | |  | |
|  |  |  |  |  |  |  |  |  |  |  |  |  |  | |
| Variable | Coeff. | *SE* | *p* | *LLCI* | *ULCI* | Coeff. | | *SE* | *p* | LLCI | ULCI |  | |  |
| Group | **54.52** | **3.51** | **< 0.01** | **47.56** | **61.48** | -19.60 | | 13.42 | 0.15 | -46.19 | 6.99 |  | |  |
| Posttest HR | – | – | – | – | – | -0.24 | | 0.20 | 0.24 | -0.64 | 0.16 |  | |  |
| Pretest HR | **0.49** | **0.14** | **< 0.01** | **0.21** | **0.77** | 0.45 | | 0.32 | 0.16 | -0.19 | 1.09 |  | |  |
| Pretest P3 Latency | -0.03 | 0.03 | 0.32 | -0.10 | 0.03 | **0.82** | | **0.07** | **< 0.01** | **0.68** | **0.96** |  | |  |
| Constant | **46.96** | **16.95** | **0.01** | **13.40** | **80.53** | 56.34 | | 38.11 | 0.14 | -19.15 | 131.84 |  | |  |
|  | *R*^2^ = 0.74 | | |  |  | *R*^2^ = 0.56 | | | |  |  |  | |  |
|  | *F*(3,117) = 111.13, *p* <0.01 | | | | | | *F*(4,116) = 36.84, *p* < 0.01 | | | | | |  | |
| Indirect effect of Group on Posttest Flanker P3 Latency | | | | | | | | | | | | |  | |
|  | *Effect* | *SE* |  | *LLCI* | *ULCI* |  | |  |  |  |  |  | |  |
| Posttest HR | -13.10 | 9.77 |  | -32.77 | 5.43 |  | |  |  |  |  |  | |  |
| Results of regression analysis accounting for pretest Flanker P3 latency and physiological variables, modeling physiological variable (for estimating *a*) and posttest Flanker P3 latency (for estimating *b*). Significant direct and indirect (*a × b*) effects are **bolded** for clarity. RT = response time, HR = heart rate, SE = standard error. Lower limit (LLCI) and upper limit (ULCI) 95% confidence intervals were calculated based on 5,000 bootstrap samples. | | | | | | | | | | | | |  | |
