# Supplementary material for: Brawn and Brainpower: Acute Resistance Exercise Improves Behavioral and Neuroelectric Measures of Executive Function
Source: Psychophysiology. 2025 Oct 30;62(11):e70171. doi: 10.1111/psyp.70171 (PMC12575885; doi:10.1111/psyp.70171)
Supplement: Supplementary file 8 — Table S4: N‐back response time mediation analyses. [file PSYP-62-e70171-s006.docx]

| **Table S4. N-back Response Time Mediation Analyses** | | | | | | | | | | |  |
| --- | --- | --- | --- | --- | --- | --- | --- | --- | --- | --- | --- |
| *n* = 114 | Outcome Variable | | | | | | | | | |  |
|  | Posttest Systolic Blood Pressure | | | | | Posttest Nback Response Time | | | | |  |
|  |  |  |  |  |  |  |  |  |  |  |  |
| Variable | Coeff. | *SE* | *p* | *LLCI* | *ULCI* | Coeff. | *SE* | *p* | LLCI | ULCI |  |
| Group | **17.63** | **1.89** | **< 0.01** | **13.87** | **21.38** | -14.92 | 20.50 | 0.47 | -55.55 | 25.70 |  |
| Posttest Systolic | – | – | – | – | – | **-1.60** | **0.77** | **0.04** | **-3.13** | **-0.07** |  |
| Pretest Systolic | **0.89** | **0.07** | **< 0.01** | **0.75** | **1.04** | 0.89 | 0.90 | 0.33 | -0.90 | 2.68 |  |
| Pretest Nback RT | 0.01 | 0.01 | 0.36 | -0.01 | 0.02 | **0.75** | **0.05** | **< 0.01** | **0.65** | **0.84** |  |
| Constant | 7.85 | 10.05 | 0.44 | -12.08 | 27.77 | **237.65** | **81.42** | **< 0.01** | **76.27** | **399.03** |  |
|  | *R*^2^ = 0.72 | | |  |  | *R*^2^ = 0.72 | | |  |  |  |
|  | *F*(3,110) = 91.57, *p* <0.01 | | | | | *F*(4,109) = 69.08, *p* < 0.01 | | | | |  |
| Indirect effect of Group on Posttest Nback RT | | | | | | | | | | |  |
|  | *Effect* | *SE* |  | *LLCI* | *ULCI* |  |  |  |  |  |  |
| Posttest Systolic | **-28.20** | **12.38** |  | **-53.22** | **-5.37** |  |  |  |  |  |  |
| *n* = 114 | Outcome Variable | | | | | | | | | |  |
|  | Posttest Diastolic Blood Pressure | | | | | Posttest Nback Response Time | | | | |  |
|  |  |  |  |  |  |  |  |  |  |  |  |
| Variable | Coeff. | *SE* | *p* | *LLCI* | *ULCI* | Coeff. | *SE* | *p* | LLCI | ULCI |  |
| Group | **-3.06** | **1.27** | **0.02** | **-5.58** | **-0.54** | **-46.72** | **15.81** | **< 0.01** | **-78.06** | **-15.38** |  |
| Posttest Diastolic | – | – | – | – | – | -1.24 | 1.16 | 0.29 | -3.54 | 1.07 |  |
| Pretest Diastolic | **0.73** | **0.06** | **< 0.01** | **0.60** | **0.86** | -0.12 | 1.16 | 0.91 | -2.42 | 2.17 |  |
| Pretest Nback RT | 0.00 | 0.00 | 0.43 | -0.01 | 0.01 | **0.74** | **0.05** | **< 0.01** | **0.65** | **0.83** |  |
| Constant | **20.10** | **5.59** | **< 0.01** | **9.03** | **31.18** | **257.86** | **71.73** | **< 0.01** | **115.68** | **400.03** |  |
|  | *R*^2^ = 0.55 | | |  |  | *R*^2^ = 0.71 | | |  |  |  |
|  | *F*(3,110) = 43.44, *p* <0.01 | | | | | *F*(4,109) = 67.14, *p* < 0.01 | | | | |  |
| Indirect effect of Group on Posttest Nback RT | | | | | | | | | | |  |
|  | *Effect* | *SE* |  | *LLCI* | *ULCI* |  |  |  |  |  |  |
| Posttest Diastolic | 3.78 | 3.75 |  | -3.15 | 12.01 |  |  |  |  |  |  |
| *n* = 114 | Outcome Variable | | | | | | | | | |  |
|  | Posttest Lactate | | | | | Posttest Nback Response Time | | | | |  |
|  |  |  |  |  |  |  |  |  |  |  |  |
| Variable | Coeff. | *SE* | *p* | *LLCI* | *ULCI* | Coeff. | *SE* | *p* | LLCI | ULCI |  |
| Group | **6.19** | **0.42** | **< 0.01** | **5.36** | **7.02** | -30.46 | 26.17 | 0.25 | -82.34 | 21.41 |  |
| Posttest Lactate | – | – | – | – | – | -2.32 | 3.44 | 0.50 | -9.15 | 4.50 |  |
| Pretest Lactate | -0.15 | 0.40 | 0.70 | -0.96 | 0.65 | -23.95 | 14.62 | 0.10 | -52.93 | 5.03 |  |
| Pretest Nback RT | 0.00 | 0.00 | 0.27 | -0.01 | 0.01 | **0.76** | **0.05** | **< 0.01** | **0.66** | **0.85** |  |
| Constant | 1.08 | 1.07 | 0.32 | -1.04 | 3.19 | **183.16** | **38.73** | **< 0.01** | **106.41** | **259.92** |  |
|  | *R*^2^ = 0.66 | | |  |  | *R*^2^ = 0.72 | | |  |  |  |
|  | *F*(3,110) = 72.32, *p* <0.01 | | | | | *F*(4,109) = 68.34, *p* < 0.01 | | | | |  |
| Indirect effect of Group on Posttest Nback RT | | | | | | | | | | |  |
|  | *Effect* | *SE* |  | *LLCI* | *ULCI* |  |  |  |  |  |  |
| Posttest Lactate | -14.38 | 17.40 |  | -48.43 | 19.88 |  |  |  |  |  |  |
| *n* = 114 | Outcome Variable | | | | | | | | | |  |
|  | Posttest Heart Rate | | | | | Posttest Nback Response Time | | | | |  |
|  |  |  |  |  |  |  |  |  |  |  |  |
| Variable | Coeff. | *SE* | *p* | *LLCI* | *ULCI* | Coeff. | *SE* | *p* | LLCI | ULCI |  |
| Group | **54.17** | **3.55** | **< 0.01** | **47.12** | **61.21** | -23.82 | 29.23 | 0.42 | -81.76 | 34.12 |  |
| Posttest HR | – | – | – | – | – | -0.43 | 0.45 | 0.33 | -1.32 | 0.45 |  |
| Pretest HR | **0.47** | **0.14** | **< 0.01** | **0.19** | **0.76** | 0.43 | 0.70 | 0.54 | -0.95 | 1.82 |  |
| Pretest Nback RT | 0.02 | 0.01 | 0.12 | 0.00 | 0.04 | **0.75** | **0.05** | **< 0.01** | **0.66** | **0.84** |  |
| Constant | 23.14 | 13.01 | 0.08 | -2.65 | 48.93 | **150.75** | **61.36** | **0.02** | **29.12** | **272.38** |  |
|  | *R*^2^ = 0.75 | | |  |  | *R*^2^ = 0.71 | | |  |  |  |
|  | *F*(3,110) = 106.14, *p* <0.01 | | | | | *F*(4,109) = 65.89, *p* < 0.01 | | | | |  |
| Indirect effect of Group on Posttest Nback RT | | | | | | | | | | |  |
|  | *Effect* | *SE* |  | *LLCI* | *ULCI* |  |  |  |  |  |  |
| Posttest HR | -23.50 | 24.07 |  | -71.24 | 23.94 |  |  |  |  |  |  |
| Results of regression analysis accounting for pretest N-back response time and physiological variables, modeling physiological variable (for estimating a) and posttest N-back response time (for estimating b). Significant direct and indirect (a × b) effects are **bolded** for clarity. RT = response time, HR = heart rate, SE = standard error. Lower limit (LLCI) and upper limit (ULCI) 95% confidence intervals were calculated based on 5,000 bootstrap samples. | | | | | | | | | | |  |
